# Roles of insect odorant binding proteins in communication and xenobiotic adaptation

**DOI:** 10.3389/finsc.2023.1274197

**Published:** 2023-10-06

**Authors:** James A. Abendroth, Timothy W. Moural, Hongshuang Wei, Fang Zhu

**Affiliations:** ^1^ Department of Entomology, Pennsylvania State University, University Park, PA, United States; ^2^ Institute of Medicinal Plant Development, Chinese Academy of Medical Sciences and Peking Union Medical College, Beijing, China; ^3^ Huck Institutes of the Life Sciences, Pennsylvania State University, University Park, PA, United States

**Keywords:** xenobiotics, semiochemicals, adaptation, co-option, host location, pesticide resistance

## Abstract

Odorant binding proteins (OBPs) are small water-soluble proteins mainly associated with olfaction, facilitating the transport of odorant molecules to their relevant receptors in the sensillum lymph. While traditionally considered essential for olfaction, recent research has revealed that OBPs are engaged in a diverse range of physiological functions in modulating chemical communication and defense. Over the past 10 years, emerging evidence suggests that OBPs play vital roles in purifying the perireceptor space from unwanted xenobiotics including plant volatiles and pesticides, potentially facilitating xenobiotic adaptation, such as host location, adaptation, and pesticide resistance. This multifunctionality can be attributed, in part, to their structural variability and effectiveness in transporting, sequestering, and concealing numerous hydrophobic molecules. Here, we firstly overviewed the classification and structural properties of OBPs in diverse insect orders. Subsequently, we discussed the myriad of functional roles of insect OBPs in communication and their adaptation to xenobiotics. By synthesizing the current knowledge in this field, our review paper contributes to a comprehensive understanding of the significance of insect OBPs in chemical ecology, xenobiotic adaptation, paving the way for future research in this fascinating area of study.

## 1 Introduction

**Figure 1:**
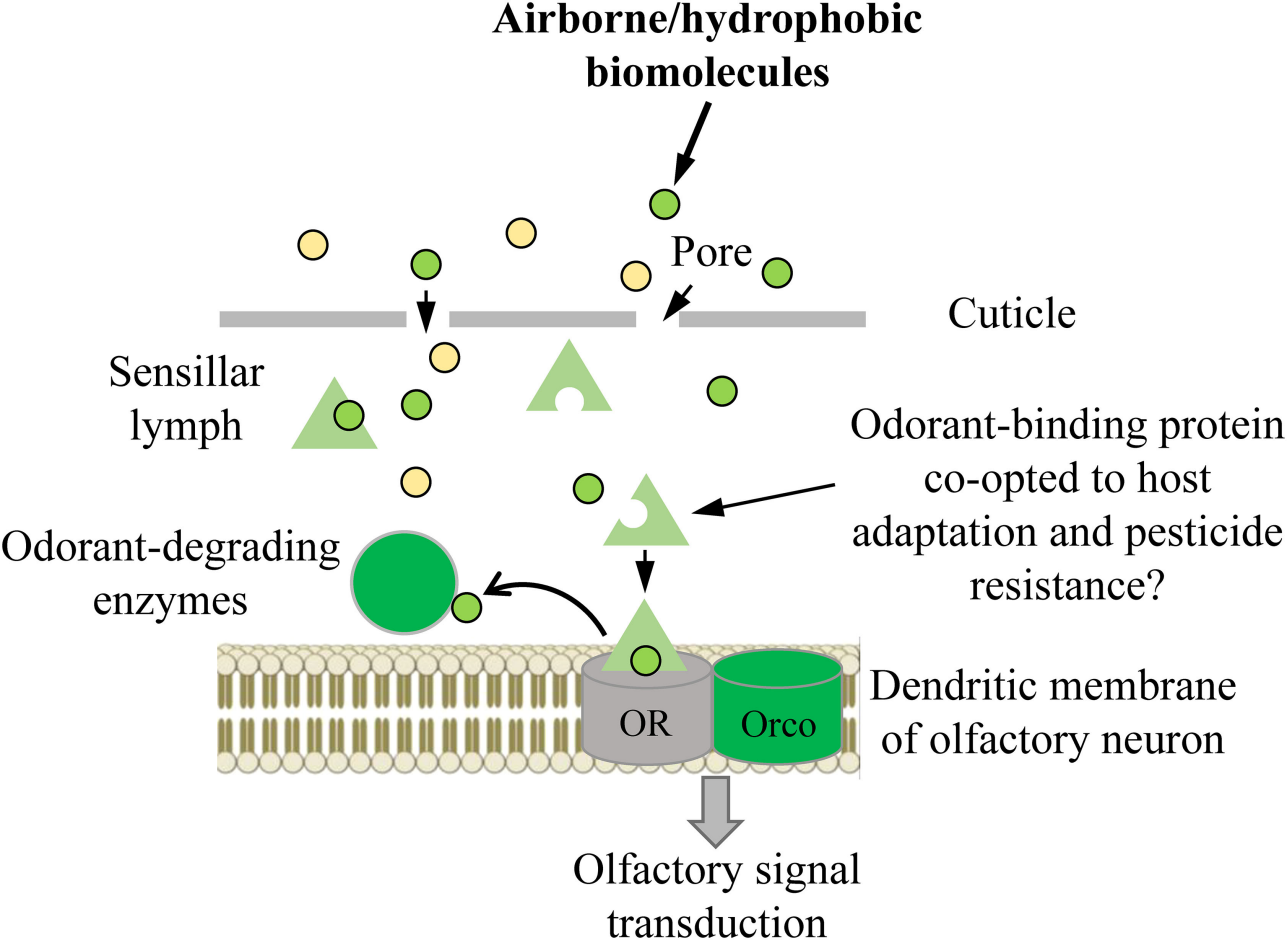
Schematic summary of the odor path. OR, odorant receptor (in some cases, it can involve other olfactory receptors, such as ionotropic receptors); Orco, co-receptor for OR.

The ability to perceive and differentiate various chemical stimuli present in a set environment is paramount to an organism’s success (1–4). Insects, the most successful group of animals on Earth, have developed a sophisticated olfactory system that has widely contributed to this success. Insect olfactory systems are known for their remarkable sensitivity and the ability to integrate odorant blends through distributed specificity of receptor tuning profiles (5–7). The classification and integration of these profiles in different portions of “odor space” rely on structures like the mushroom body and lateral horn of the protocerebrum, enabling precise discrimination of pheromone blends or subtle differences in plant odor blends (5, 8). Insect olfaction is composed of several transmembrane receptors and soluble and insoluble proteins, which collaborate harmoniously to receive, process, interpret, and ultimately react to external stimuli (3). The key olfactory proteins involved in this process include odorant binding proteins (OBPs), odorant receptors (ORs), ionotropic receptors (IRs), odorant degrading enzymes (ODEs). and sensory neuron membrane proteins (SNMPs) (3). ORs form a heteromeric complex with a ubiquitous coreceptor coined odorant receptor co-receptor (Orco) that is omni-present in every functional OR complex and is highly conserved among all insects (3). In general, exogenous odorants or volatiles enter the sensillum lymph through cuticular pores and are subsequently bound and solubilized by OBPs, wherein this OBP-odorant complex is transported across the sensillum to a candidate OR for transduction (3, 9) (**Figure 1**). Once the OBP-odorant complex (or the odorant alone) is bound to a receptive OR, a transduction cascade is triggered, which leads to action potentials transmitting from olfactory receptor neurons to the higher integration centers within the protocerebrum. Odorants must be deactivated rapidly by ODEs or scavengers once this occurs, otherwise efficiency of olfactory processes will be impaired via prolonged exposure of the respective odorant inducing overstimulation. Numerous lines of evidence suggest that many ODEs such as cytochrome P450s, glutathione S-transferases (GSTs), carboxyl/cholinesterases (CCEs) are involved in degrading volatile molecules during the deactivation process (3, 10–13). Some studies indicate that prior to degradation by ODEs, pheromones undergo deactivation through their binding to OBPs (e.g., pheromone binding proteins, PBPs). Additionally, these OBPs serve as scavengers, contributing to the decline of the receptor potential after stimulus offset. This implies the existence of a broader molecular mechanism beyond enzymatic degradation (3, 14–17).

Within the realm of olfaction processing, OBPs play a vital role as the primary mediators connecting the external environment with ORs (7, 9). OBPs are frequently necessary for safeguarding exogenous hydrophobic volatiles against degradation prior to their interaction with the corresponding ORs. This protection occurs following the initial uptake, binding, and transportation of these volatiles within the aqueous sensillum lymph. The delivery of the exogenous volatiles to the OR triggers an elicited response, allowing for the recognition of volatiles from hosts or natural enemies and identification of pheromones of potential mates. Following the stimulation of ORs by exogenous molecules, OBPs may also participate as molecular traps, preventing neuron oversaturation (1–3, 17–20). In addition, evidence shows that OBPs may play essential roles in cleaning the perireceptor space from undesirable xenobiotics, including plant volatiles and pesticides. This function potentially contributes to host plant adaptation and pesticide resistance (20–27). Despite their primary role as olfactory proteins, recent research has identified OBPs to be involved in a variety of physiological roles in insects outside of olfactory tissues, owing in part to their structural variability and efficacy in the transporting, sequestering, and concealing of various hydrophobic molecules (2, 3, 9, 28–30).

Roughly half of insect species are phytophagous, forming a close relationship with the host plants they feed and interact with (31). During the coevolution of insects and plants over hundreds of millions of years, insects have evolved diverse mechanisms to adapt to numerous xenobiotics (12, 13, 32–34). Olfaction in insects may serve as an “Achilles heel” - a target for plant defense because of its remarkable sensitivity, critical importance, and vulnerability (22). OBPs serve as the primary point of contact for the insect olfactory system with xenobiotics, playing a principal role in modulating chemical communication and defense. Here, we initially summarize the classification and structural properties of OBPs in various insect orders. Then we focus on the variety of functional roles of OBPs in insect communication and adaptation to xenobiotics. Our review concludes with prospective thoughts on future studies that could expand our knowledge of OBPs and their diverse functions in chemical ecology and xenobiotic adaptation.

## 2 Classification and structural characteristics of insect OBPs

**Figure 2:**
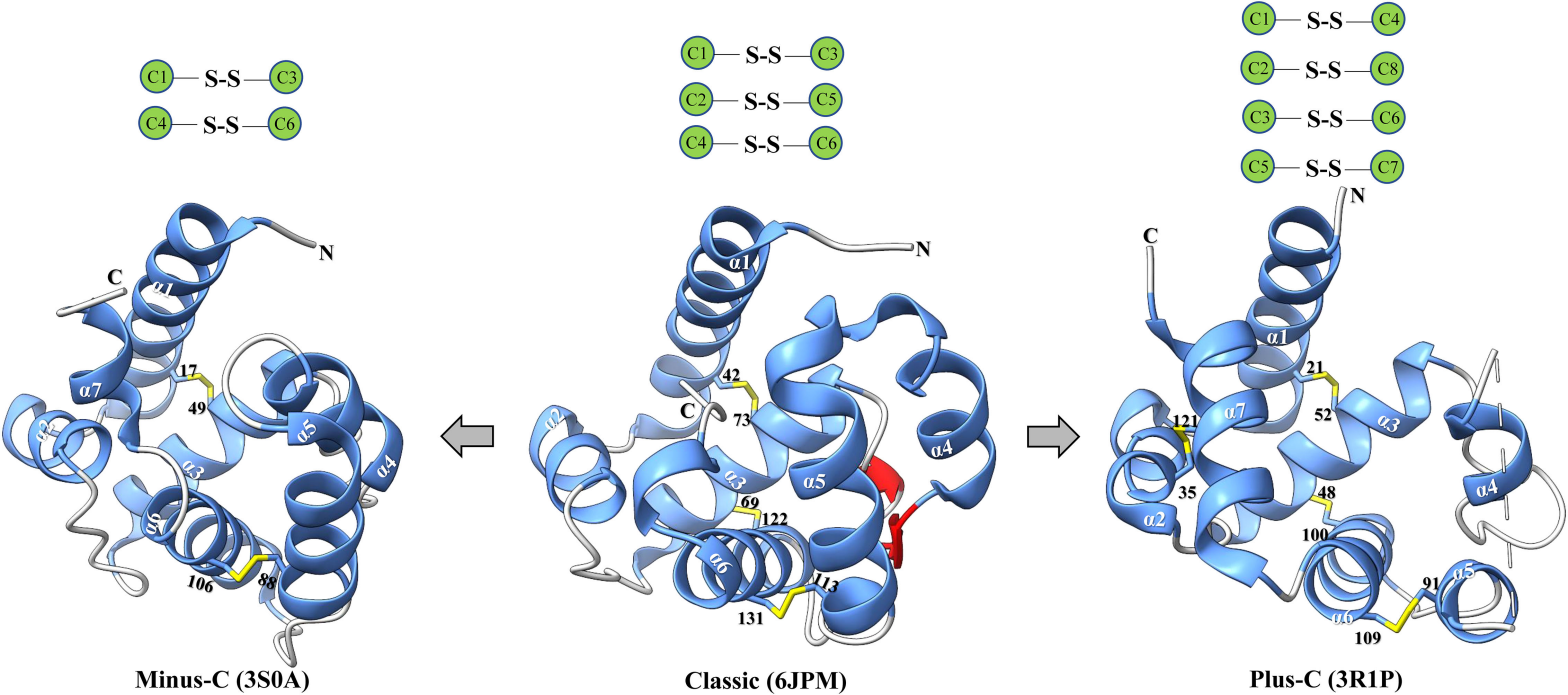
Major classes of insect OBPs. Beginning from the left, minus-C (e.g. *Apis mellifera* AmelOBP14, PDB ID:3S0A); Classic (e.g. *Chrysopa pallens* CpalOBP4, PDB ID:6JPM); Plus-C (e.g. *Anopheles gambiae* AgamOBP7, PDB ID:3R1P). Blue indicates α-helices; yellow indicates disulfide bridge; red indicates strands; and lastly grey indicates coils. Black text indicates a conserved cysteine residue, white text indicates an α-helix. Below each protein is the corresponding class of the odorant binding protein and the protein database reference used to generate the specific protein. Three-dimensional protein structures were constructed using the program ChimeraX.

**Figure 3:**
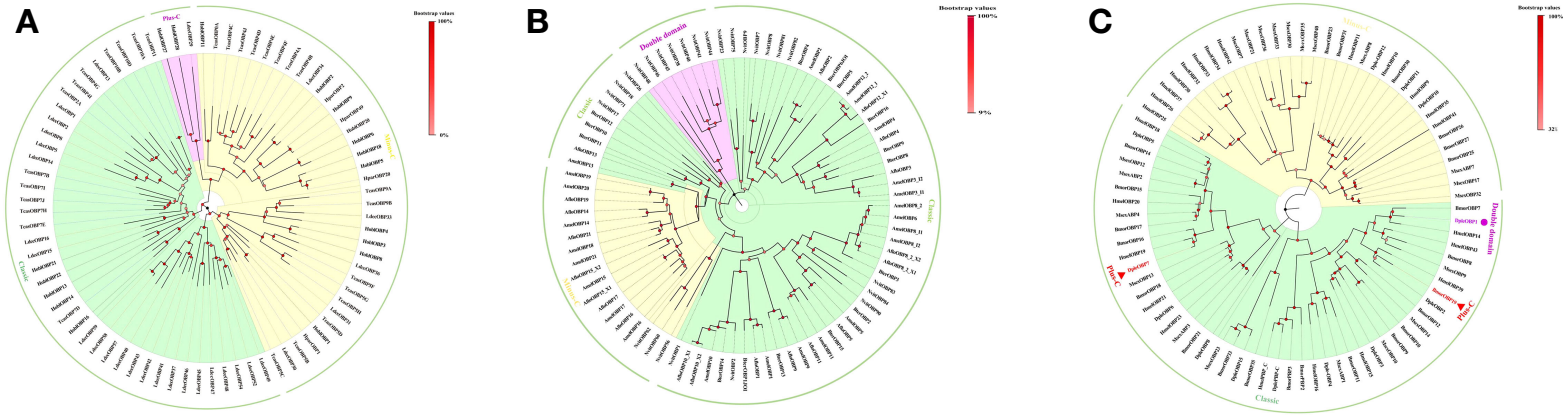
Phylogenetic analysis of insect OBPs in three major orders, and OBPs used in the analysis had been previously characterized through either proteomic or transcriptomic analyses. **(A)** Coleopteran insect OBPs from *Holotrichia oblita, Holotrichia parallela, Leptinotarsa decemlineata*, and *Tribolium castaneum*; **(B)** Hymenopteran insect OBPs from *Apis florea, Apis melifera, Bombus terrestris, and nasiona vitripennis*; **(C)** Lepidopteran insect OBPs from *Bombyx mori, Danaus plexippus, Heliconius melpomene*, and *Manduca sexta*. Phylogenetic trees were inferred by the neighbor-joining method (37) and were created using MEGA11 software (38). The trees were visualized using Figtree v1.4.4 software.

Insect OBPs are small water-soluble extracellular proteins, ranging from between roughly 100 to ~200 amino acid residues, with very little sequence similarity within OBPs of the same species (1). Initially described in Lepidoptera (16), these proteins were categorized into three separate subfamilies based on the amino acid sequences and differential expression patterns: pheromone binding proteins (PBPs), general odorant binding proteins (GOBPs), and antennal binding proteins (ABPs) (1, 16). However, a primary challenge with this classification methods arises from the significant variation observed in the amino acid sequences, ligand binding affinity, differential expression, and functional roles beyond Lepidoptera, extending even to functions beyond chemosensation (35, 36). Therefore, there was a pressing need for a more comprehensive and flexible classification method to accurately characterize their diverse functional roles and implications. Currently, insect OBPs are generally divided into three primary groups based on the number of conserved cysteine residues and interlocked disulfide bridges: 1) Classic OBPs (e.g. *Chrysopa pallens* CpalOBP4, PDB ID:6JPM), which have six conserved cysteine residues that participate in three disulfide bridges; 2) Minus-C OBPs (e.g. *Apis mellifera* AmelOBP14, PDB ID:3S0A), featuring four or five conserved cysteine residues and two disulfide bridges; 3) Plus-C OBPs (e.g. *Anopheles gambiae* AgamOBP7, PDB ID:3R1P), which possess eight or more conserved cysteine residues, four or more disulfide bridges, and a conserved proline residue (**Figure 2**) (36). Among these groups, Classic OBPs are the most frequently identified type of OBPs in every insect genome (**Table 1**; **Figures 2**, **3**). Phylogenic analysis of insect OBPs have shown that Classic OBPs seem to be the basal group, and other Minus-C and Plus-C groups of OBPs are subgroups of the Classic OBPs (39). This may suggest that Minus-C and Plus-C OBPs likely diverged from the Classic OBPs (39–41) (**Figure 2**). However, the relative composition of OBPs in an insect genome can vary greatly, as some OBP groups may feature a larger expansion in one group of insects as compared to others, as has been observed in certain beetle species (35, 42–47) (**Figure 3A**; **Table 1**). There is a group of OBPs that has been termed “atypical OBPs” characterized by 10 or more conserved cysteines, a long C-terminus, a conserved proline residue, and four or more disulfide bridges, which is recorded in several mosquito and locust species, suggesting this group of genes may be recently evolved in these species (36, 48–50). Additionally, groups of insect OBPs that exist outside of the three primary structural groups can be found in certain insects, such as double domain OBPs that are found exclusively in certain wasp species (51) and Dimer OBPs that are found in some species of dipterans and lepidopterans (**Figures 3A–C**; **Table 1**) (39, 51). In certain insect groups, there is a complete absence of an entire primary group of OBPs; for instance, honey bees lack of plus-C OBPs all together (**Figure 3B**; **Table 1**) (40). The amount of OBP genes in an insect genome can vary greatly among species, ranging from as low as 7 in *Ceratosolen solmsi* to as high as 111 in *Aedes aegypti* (**Table 1**). The reason why certain insect species possess a higher number of OBPs while others have relatively few remains unclear. However, this disparity can likely be attributed to the insects’ unique lifestyles, evolutionary processes, and wide variety of environments (39).

**Table 1:** Number of Odorant Binding Protein genes and classification in genomes or transcriptomes of 37 insect species.

Order	Species	Total	Classic	Minus-C	Plus-C	Other*	Reference ^$^
**Blattodea**	*Blatella germanica*	109	38		71	0	(1)
	*Periplaneta americana* ^†^	60	37	3	20	0	(2)
	*Zootermopsis nevadensis*	29	19	3	7	0	(2)
**Coleoptera**	*Anoplophora glabripennis*	52	20	31	1	0	(3)
	*Dendroctonus ponderosae*	31	18	12	1	0	(4)
	*Holotrichia oblita* ^†#^	29	19	7	3	0	(5)
	*Holotrichia parallela* ^†#^	25	15	6	4	0	(6)
	*Leptinotarsa decemlineata^#^*	59	14	43	1	1	(7)
	*Tenebrio molitor* ^†^	19	10	8	0	1	(8)
	*Tribolium castaneum^#^*	49	20	21	1	7	(9, 10)
**Diptera**	*Aedes aegypti*	111	39	0	27	45	(11)
	*Anopheles gambiae*	69	29	0	20	20	(11)
	*Anopheles stephensi*	44	27	0	7	10	(12)
	*Culex quinquefasciatus*	109	69	0	12	28	(11)
	*Drosophila melanogaster*	52	28	7	15	2	(13-15)
**Hemiptera**	*Acyrthosiphon pisum*	15	13	0	2	0	(16)
	*Adelphocoris lineolatus* ^†^	14	12	0	2	0	(17)
	*Bemisia tabaci*	8	5	1	2	0	(18)
	*Riptortus pedestris*	49	41	0	8	0	(19)
	*Tropidothorax elegans* ^†^	19	14	0	5	0	(20)
**Hymenoptera**	*Aphidius gifuensis* ^†^	14	12	2	0	0	(21)
	*Apis florea^#^*	22	13	9	0	0	(22)
	*Apis mellifera^#^*	21	13	8	0	0	(22, 23)
	*Bombus terrestris^#^*	16	16	0	0	0	(24)
	*Ceratosolen solmsi*	7	7	0	0	0	(25, 26)
	*Cotesia vestalis*	20	18	2	0	0	(27, 28)
	*Nasiona vitripennis^#^*	90	72	8	0	10**	(29)
**Lepidoptera**	*Bombyx mori^#^*	44	29	9	6	0	(29, 30)
	*Danaus plexippus^#^*	32	19	6	6	1	(31)
	*Heliconius Melpomene^#^*	51	23	22	6	0	(31)
	*Manduca sexta^#^*	49	24	18	7	0	(31)
	*Plutella xylostella*	39	39	0	0	0	(32)
	*Spodoptera frugiperda*	33	25	3	3	2	(33)
**Orthoptera**	*Locusta migratoria*	17	11	0	5	1	(34)
	*Oedaleus asiaticus* ^†^	15	10	1	4	0	(35)
	*Schistocerca gregaria* ^†^	14	9	0	3	2	(35)
**Thysanoptera**	*Odontothrips loti* ^†^	7	5	1	0	1	(36)

^^†^^stands for the data collected from transcriptome studies; ^*^ "Other" corresponds to unidentified OBPs or OBPs that do not fall under the classic, minus-C, and plus-C classification; ^**^ These OBPs are minus-C OBPs, but possess a double domain in their sequence, as compared to typical minus-C OBPs in other insect species; ^$^ These references are listed in the **Supplementary Material**; ^#^ OBPs from these species were used in the generation of the phylogenetic trees featured in **Figure 3**.

Despite the high diversity and variation among insect OBPs, this group of proteins has some hallmark features. In addition to the extremely conserved cysteine residues, insect OBPs typically have two to four interconnected disulfide bridges (e.g., a pattern of C1-C3, C2-C5, and C4-C6) that play a vital role in stabilizing the protein (52–58) (**Figure 2**). Furthermore, six α-helices, which may vary in number in certain cases, synergistically work with the interlocked disulfide bridges to further enhance the protein’s stability. Specific α-helices may be involved in forming a hydrophobic cavity crucial for ligand binding activity (52, 53, 58–60). The ligand binding specificity of insect OBPs exhibits significant variation, ranging from high specificity to remarkable broadness. This diversity is influenced by the overall size and shape of the binding pocket, as well as the specific amino acids lining it (54, 55). Previous studies have demonstrated that variability in amino acid identity and length of the C-terminal region can influence ligand binding affinity. For example, in a specific case, the rearrangement of amino acids within the C-terminus region of a *Drosophila melanogaster* OBP (LUSH) disrupted the formation of a salt bridge, resulting in impaired binding ability to the expected ligand 11-*cis* vaccenyl acetate, a conspecific male sex pheromone (56). The length variation in the C-terminal region also impacts the interaction of the C-terminus with the hydrophobic binding cavity. Insect OBPs with longer C-terminus regions possess a flap that can cover the entrance of the binding cavity, whereas those with shorter C-terminus regions leave their binding cavities exposed to bulk solvent (2, 61, 62). Additional research has demonstrated that pH-induced conformational changes can impact the ligand-binding capability of specific insect OBPs (52, 63–65). Notably, Lepidopteran OBPs AtraPBP1 from *Amyelois transitella* and ApolPBP from *Antheraea polyphemus* possess a C-terminal region that plays a crucial role in pheromone binding and release, triggered by changes in pH levels (66, 67). In AtraPBP1, the polar amino acid residues Glu132 and Glu141 create two salt bridges with protonated histidine residues His 80 and His95, respectively. These two salt bridges are induced by acidic conditions, promoting the formation of a seventh helix at the C-terminal region that can compete with the ligand and trigger its release (9, 66). In contrast to OBPs in Lepidoptera, the majority of Dipteran OBPs lack a sufficiently long C-terminal region to form an additional helix able to occupy the binding cavity (9, 52). Nevertheless, many Dipteran OBPs, such as AaegOBP1 (*Ae. aegypti*, PDB ID:3K1E), AgamOBP1 (*An. gambiae*, PDB ID:2ERB), CquiOBP1 (*Culex quinquefasciatus*, PDB ID:3OGN), undergo pH-depended conformation changes associated with loss of binding affinity, similar to what has been observed in Lepidopteran OBPs, indicating a distinct mechanism (9). The C-terminal region of these Dipteran insect OBP proteins instead function as a “lid” over the binding cavity, a characteristic not found in other insect groups. This lid was suggested to act as a pH-sensitive hinge, moving away from the binding cavity when pH is reduced, as the OBP-odorant complex approaches the dendritic membrane (9, 52). Moreover, the ligand binding ability of an OBP may be affected by its molecular volume. For example, in the Minus-C OBP DhelOBP21 of *Dastarcus helophoroides*, the ligand being either too small (<100 A^3^) or too large (>185 A^3^) can disrupt its facultative binding ability (68). Additionally, hydrophobic and hydrogen bond interactions can also influence binding efficacy of an OBP, and the absence of either can lead to substantial reductions in the binding affinity of an OBP towards a ligand (68, 69). Lastly, it is worth noting that the majority of determined crystal structures of insect OBPs reveal a tendency for dimerization upon ligand binding (59, 70–73) (**Table 2**). Insect OBP protein structures in both ligand-free apo forms and in complex with various ligands, have been determined using protein crystallography and nuclear magnetic resonance (NMR) spectroscopy (9, 74). A list of currently published insect OBP structures at the time of this publication has been provided in **Table 2**. The list includes 27 individual insect OBP structures across 17 insect species, including 10 OBP structures from species in Diptera and 7 OBP structures from species in Lepidoptera. Currently, our understanding is limited, as over half of the elucidated structures (17 out of 27) come from Dipteran and Lepidopteran insects (**Table 2**). Further research is crucial to comprehensively understand the relationship between the varied structures and functions of numerous OBPs from a wide range of insect species.

**Table 2:** List of 27 three-dimensional crystal structures, classification, and function of insect Odorant Binding Proteins.

Order	Species Name	Name (PDB number)	Classification	Function	Reference^*^
Blattodea	*Leucophaea maderae*	PBP (1ORG)	Classic	Involved in recognition of sex pheromone components: 3-hydroxy-butan-2-on and butane-2,3-diol	(37, 38)
Coleoptera	*Tenebrio molitor*	THP12 (1C3Z)	Minus-C	N/A	(39)
Diptera	*Aedes aegypti*	OBP1 (3K1E)	Classic	N/A	(40)
		OBP22 (6OG0)	Classic	Potentially involved in the recognition of fatty acids	(41)
	*Anopheles gambiae*	OBP1 (2ERB)	Classic	Involved in host recognition	(42-45)
		OBP7 (3R1P)	Plus-C	N/A	(46)
		OBP20 (3VB1)	Classic	N/A	(47)
		OBP47 (3PM2)	Plus-C	N/A	(48)
		OBP48 (4KYN)	Plus-C	N/A	(49)
	*Culex quinquefasciatus*	OBP1 (3OGN)	Classic	Modulates ovipositional preference	(50, 51)
	*Drosophila melanogaster*	OBP28A (6QQ4)	Classic	Involved in the detection and mediation of sensitivity to fruit-like odors	(52)
		LUSH (OBP76A) (1T14)	Classic	Involved in host and pheromone recognition through mediation of alcohol compounds	(53-55)
Hemiptera	*Megoura viciae*	OBP3 (4Z39)	Classic	Potentially involved in the recognition of alarm pheromones	(56)
	*Nasovonia ribisnigri*	OBP3 (4Z45)	Classic	Potentially involved in the recognition of alarm pheromones	(56)
Hymenoptera	*Apis melifera*	ASP1 (OBP1) (3BJH)	Classic	Involved in the recognition of the queen pheromone	(57-60)
		OBP5 (3R72)	Classic	N/A	To be published
		ASP2 (GOBP2) (1TUJ)	Classic	Involved in non-sexual pheromone recognition	To be published, (61, 62)
		OBP14 (3S0A)	Minus-C	Binds with the highest affinity to citralva and eugenol	(63)
Lepidoptera	*Amyelois transitella*	PBP1 (2KPH)	Classic	Involved in the recognition and transport of non-polar pheromone	(64, 65)
	*Antheraea polyphemus*	PBP1 (1QWV)	Classic	Involved in the recognition of sex pheromone component (E, Z)-6,11-hexadecadienyl acetate (AC1)	(66-69)
	*Bombyx mori*	PBP1 (1DQE)	Classic	Modulates sensitivity to the sex pheromone bombykol	(70-72)
		GOBP2 (2WC5)	Classic	Involved in the recognition and discrimination of the sex pheromones bombykol and bombykal	(73, 74)
	*Epiphyas postvittana*	PBP3 (6VQ5)	Classic	Involved in recognition of sex pheromone components: E11-14: OAc and E9, E11-14: OAc	(75)
	*Helicoverpa armigera*	PBP1 (7VW8)	Classic	Involved in recognition of sex pheromone components: to Z11-16: Ald and Z9-16: Ald	(76, 77)
	*Lymantria dispar*	PBP1 (6UM9)	Classic	N/A	(78)
Neuroptera	*Chrysopa pallens*	OBP4 (6JPM)	Classic	Involved in the recognition of prey host plant volatiles	(79, 80)
Orthoptera	*Locusta migratoria*	OBP1 (4PT1)	Classic	N/A	(81)

PDB, protein database; N/A, not available; OaC, acetoxy functional group; Ald, aldehyde functional group. ^*^ These references are listed in the **Supplementary Material**.

## 3 Diverse roles of insect OBPs in communication and xenobiotic adaptation

Insects encounter a diverse array of semiochemicals and xenobiotics in their environment, necessitating adaptive responses. These chemicals range from allospecific and conspecific pheromones, plant allelochemicals, volatiles, and a multitude of anthropogenic compounds, such as pesticides (34, 75–77). On one hand, insects use these chemical cues to detect their food, mates, and other substrates critical for their survival and reproduction. On the other hand, insects must evolve adaptation strategies to cope with “delicious poisons”, which are harmful compounds disguised as attractants. These chemical cues can be exploited by host plants as a defensive measure, posing survival challenges for insects (22, 78). Recent studies have demonstrated that insect OBPs play critical roles in the uptake or release of a diverse spectrum of molecules due to their stable and compact structure, high variability in binding affinity, and efficiency transportation of hydrophobic molecules (79–81). Additionally, many proteomic and transcriptomic studies focusing solely on olfactory organs, such as antennae or maxillary palps, may not identify all OBP-encoding genes within an insect genome. This suggests that certain OBPs could be exclusively expressed in non-olfactory organs and/or appendages (2, 82–85). Recently, there are many integrative reviews of insect OBPs discussing their diverse expression and functions in chemoreception and beyond (1, 2, 9, 36, 74). Therefore, in this section, our focus will be on the roles of insect OBPs in communication, host location, and their co-opted functions in pesticide adaptation.

### 3.1 Pheromone detection and release

Detection of conspecific and allospecific pheromones are essential to reproductive success, survival, and overall fitness of an insect (2, 86–88). Several studies have demonstrated the role and significance of OBPs in the detection and sensitivity to pheromones across a variety of insect orders (36, 89–94) since their initial discovery in the male silk moth, by Vogt and Riddiford in 1981 (16). For example, *Bombyx mori* BmorPBP1 was suggested to be essential for the activation of the receptor *B. mori* BmorOR1 to the female released sex pheromone bombykol rather than bombykal (95–97). In the absence of BmorPBP1, only low sensitivity to bombykol was detected in transgenic *drosophila* expressing BmorOR1, however, high sensitivity and ligand specificity towards bombykol was observed in mutants expressing both BmorOR1 and BmorPBP1 (96). The affinity of BmorPBP1 to bombykol is regulated by pH-dependent conformational changes in PBP, which lead to the release of pheromones under acidic environment surrounding the OR neurons (64, 65, 89, 98). Besides BmorPBP1, conformational changes that are integral to pheromone recognition were also observed in PBPs of several other insect species (66, 99, 100). For example, in *D. melanogaster*, it was observed that LUSH PBP detects and releases the male specific sex pheromone 11-*cis*-vaccenyl acetate (cVA) to activate *D. melanogaster* OR67d neurons, linking pheromone-induced behavior with PBP-dependent activation of olfactory neurons (56, 101, 102). Additional studies demonstrated that *D. melanogaster* OBP56h influences male courtship behavior. It plays a dual role in the production of precursors to cuticular pheromones, as its expression level is linked to the expression levels of several biosynthesis enzymes (1, 103, 104). One of these cuticular pheromones, 5-tricosene, is highly expressed in males and can decrease copulation latency at high levels, potentially preventing incidences of male-male courtship (1). In *Ap. mellifera*, brood pheromone (β-ocimene) and death pheromone (oleic acid) are strong ligands for two OBPs, AmelOBP16 and AmelOBP18. Expression levels of both OBPs were found to be linked with the degree of hygienicity displayed in bee colonies, suggesting these two OBPs may play important roles in triggering honey bee hygienic behavior (105, 106). Additionally, it was found that *Ap. mellifera* AmelASP1 and *Ap. cerana* AcerOBP1 are involved in the recognition of honeybee queen pheromone (107, 108). Recently, conserved insect OBPs were identified from various aphid species and their eavesdropping predators, such as ladybird beetles, lacewings, and the marmalade hoverfly, demonstrating the potential functions of OBPs in predator-prey interactions (109–112). These OBPs play roles in detection of (E)-β-farnesene (EBF), which is the primary alarm pheromone active component in many aphid species (Hemiptera: Aphididae) and is used as chemical cue to signal danger (113–117). For example, in *Acyrthosiphon pisum*, knockdowns of *ApisOBP3* and *ApisOBP7*, that are known to bind EBF, led to the disappearance of repellent behavior caused by EBF (110, 115). The functions of related ApisOBP3 and/or ApisOBP7 proteins in EBF detection were also characterized in other aphid species by using behavioral assays, ligand-binding assays, or X-ray crystal structure examination (110, 111, 114, 118). In *Rhopalosiphum padi*, both RpadOBP3 and RpadOBP7 bound EBF and additionally, RpadOBP3 showed affinity for the ligands, EBF and several other plant volatiles, while RpadOBP7 was specific to EBF (114). Most recently, four antennae specific OBPs were functionally characterized in the aphid natural enemy, *Harmonia axyridis*. Among these OBPs, HaxyOBP15 showed a broader binding profile among various substances, including EBF and other volatiles (117). Similarly, two lacewing species OBPs, *Chrysoperla sinica* CsinOBP1 and *Chrysopa pallens* CpalOBP10, were also found to bind to EBF (112, 119).

It has been demonstrated that besides the antennae, OBPs can also be expressed in the sex glands and various other organs, participating in both the uptake and release of various pheromones. A study performed in the diving beetle *Cybister japonicus* found two OBPs specifically expressed in the foreleg and testis of male beetles, which are used for holding a female during courtship and mating, suggesting potential roles of these OBPs in chemical communication (120). The sex pheromone for this species is still unknown, therefore, further research is required to confirm the functions of these OBPs in pheromone recognition and secretion (120). Several studies have also found the presence of OBPs in the seminal fluid of a wide range of insect taxa, that are transferred to females during mating or are potentially used as oviposition deterrents on fertilized eggs (121–126). Interestingly, fruit flies possess OBPs in the seminal receptacle along with an odorant receptor, displaying the highly adaptable nature of OBPs in the insect body (121, 127). In a Lepidopteran species, *Helicoverpa armigera*, *HarmOBP10* was expressed in antennal and reproductive organs of both sexes, binding to 1-dodecene, a compound reported as an insect repellent as well as several volatile compounds, suggesting its dual roles in chemical detection and a carrier for oviposition deterrents (125).

### 3.2 Host location and adaptation

Recognition of odorants that are associated with an insect’s host is essential for locating nutrients and ultimately reproductive success (128–130). A living host of a particular insect can vary greatly based on its life history and feeding guilds, ranging from plants to other animals or humans. Insect OBPs involved in the recognition of host semiochemicals are mainly expressed in the sensillum lymph of the antennae and assist in the adaptation of an insect to their hosts, which has been demonstrated across a diverse range of taxa (131–133). For example, it was found that *An. gambiae* AgamOBP1 is involved in the recognition and sensitivity of indole and 3-methyl indole in the antennae, the former aiding in the location of a human blood host and the latter acting as an oviposition attractant (20, 134–136). Female *A. gambiae* subjected to RNAi mediated silencing of *AgamOBP1* caused a significant reduction in the ability to perceive indole, some individuals even exhibiting a complete loss of perception (136). Another study demonstrated that *Drosophila sechellia* OBP57d and OBP57e are involved in modulating the differences in taste perception and behavioral response towards its host plant *Morinda citrifolia* (28). The characteristic odor of the ripe fruit is due to the compounds hexanoic acid and octanoic acid, that have been shown to induce a repellent effect and cause mortality in other *Drosophila* species (137). After inducing the knockdown of *OBP57d* and *OBP57e* in *D. melanogaster*, it was found that the prior repellent behavior towards ripe fruit was replaced with attraction, suggesting that both OBPs participate in the adaptation of *Drosophila* to a toxic host (28, 138). In another study, it was found that *Nilapavarta lugens* NlugOBP11 is secreted during feeding on rice and alters upregulation of the plant phytohormone salicylic acid in the brown planthopper (139). Silencing of *NlugOBP11* expression resulted in a decrease in feeding performance and eventual death, but overexpression of *NlugOBP11* in the protoplast of rice suppressed the expression of salicylic acid genes, suggesting the contribution of NlugOBP11 in host plant adaptation. In contrast to prior reports, a recent study has shown that host semiochemicals can induce an opposite effect in an insect in the absence of certain OBPs (140). After RNAi-mediated silencing of *D. helophoroides DhelOBP4*, compounds that previously elicited a strong attractant response induced a sexually dimorphic inverse effect in this ectoparasitic insect (140). Adult males no longer elicited a behavioral response and adult females exhibited a strong repellent to the herbivore induced plant volatiles, γ-terpinene and p-cymene. Although the molecular mechanism was not determined, these results may indicate the involvement of DhelOBP4 in host plant volatile recognition and/or protection of olfactory processes from potential damage by plant volatiles (140).

During the evolution of plants and phytophagous insects, plant volatiles were used as a defensive strategy to repel these insects and/or attract their respective parasitoids and predators (141). For phytophagous insects, plant volatiles are essential cues for food and oviposition (22). There is increasing evidence suggesting that plant volatiles can also function as mate-finding cues and/or stimulate sex pheromone release, which assist insects to find their mating partners (142, 143). Recently, more functional studies suggested it is a common phenomenon that insect OBPs can bind both sex pheromone components and plant volatiles, including green leaf and floral volatiles (80, 144–150). Competitive fluorescence binding assays, for instance, have shown that in the rice leaffolder, *Cnaphalocrocis medinalis*, CmedPBP4 could selectively recognize three sex pheromones and eleven rice plant volatiles (145). In the geometrid moth *Ectropis obliqua*, EoblPBP1 bound three sex pheromone components and several green leaf volatiles that had been demonstrated to attract virgin male *E. obliqua*, indicating that green leaf volatiles may act as synergists to enhance the efficacy of sex pheromones (147). It has also been found that some non-PBP OBPs play roles in sex pheromone recognition and plant volatile identification (144, 149–152). For example, the electroantennogram and competitive fluorescence binding assays revealed that a Classic OBP in *Phthorimaea operculella*, PopeOBP16 was involved in recognizing and binding several plant volatiles and sex pheromone components (150). In the Eastern Honeybee, *A. cerana*, two Classic OBPs, AcerOBP6 and AcerOBP11 as well as one Minus-C OBP, AcerOBP15, have been characterized and been linked to recognition of bee pheromones and floral volatiles, indicating these OBPs may play a dual-role in sensing various bee pheromones and host odorants (80, 146, 152).

### 3.3 Pesticide adaptation

Despite the remarkable sensitivity of the insect olfactory system to detect and differentiate critical odorant cues even at minute concentrations, it also can act as an attractive target for harmful plant compounds and environmental toxins (22, 24). Plant volatiles or anthropogenic toxins pose potential risks to terrestrial insects, as they can impair the processing of odorant molecules or even cause physiological damage at high doses (24). Recently, a substantial amount of evidence emerged, indicating that the gene expression of certain OBPs undergo changes in response to pesticide exposure. These OBPs may play a role in pesticide adaptation by binding, buffering, or sequestration of pesticides that have penetrated the cuticle (2, 25–27, 79, 153–159). Investigating the mechanisms underlying OBP-mediated pesticide adaptation will open new avenues to broaden our understanding of how insects adapt to their xenobiotic environment and evolution of pesticide resistance (13, 33, 160).

One of the first studies to demonstrate the potential of insect OBPs to be involved in insecticide adaptation was conducted in the diamondback moth, *Plutella xylostella* (21). The study exposed *P. xylostella* larvae to two separate selection treatment regimens: Low concentrations of permethrin (LC_5_ of prior generation) only applied to the upper and center portion of the host cabbage plants and high concentrations of permethrin (LC_50_ of prior generation) uniformly applied across the entire canopy of the cabbage plant (21). It was found that upon comparing the F_1_ parental generation to the selected G_25_ generation, *PxylOBP13* was upregulated in the low concentration of permethrin treatment group, implying a possible role in resistance. Lin et al. in 2018 reported that the gene expression of *SlituOBP9* in the tobacco cutworm *Spodoptera litura*, was increased in response to chlorpyrifos and emamectin benzoate (25). After injection of dsRNA targeting *SlituOBP9*, the survival of tobacco cutworm moths exposed to chlorpyrifos for 48 hours was decreased to 7.7%, as compared to 50% in the control moths, indicating that SlituOBP9 could play a role in chlorpyrifos adaptation (25). Similarly, it was found that exposure to the herbicide butachlor caused reduced susceptibility to chlorpyrifos in the tobacco cutworm in a separate study (156). Gene silencing of one general OBP, *S. litura SlGOBP2*, decreased larval tolerance to chlorpyrifos, suggesting that olfactory recognition of butachlor by SlGOBP2 may contribute to enhanced chlorpyrifos resistance by induction of ecdysone synthesis and regulating expressions of detoxification genes (156). In the Asian citrus psyllid, *Diaphorina citri*, the expression of *DcitOBP2* was induced in response to imidacloprid exposure. When *DcitOBP2* was silenced via RNAi, susceptibility to imidacloprid was increased in *Di. Citri* adults, suggesting that DcitOBP2 is involved in imidacloprid resistance (161). Similarly, *N. lugens* NlOBP3 was associated with nitenpyram and sulfoxaflor resistance in the brown planthopper (157). Two PBPs in *Athetis lepigone*, AlepPBP2 and AlepPBP3, had high binding affinities to an organophosphate insecticide, phoxim, indicating that these two PBPs may play roles in the phoxim adaptation of this polyphagous pest (155). Similarly, a recent study demonstrated that a G protein coupled receptor, latrophilin may contribute to insecticide resistance through regulating the expression of *Tribolium castaneum TcOBPC01* and one other chemosensory gene (27). Additionally, it was also reported that an increase in larval mortality to dichlorvos and carbofuran was observed when *latrophilin* or *TcOBPC01* was silenced.

Other than acute effects on target insect pests, chemical insecticides cause serious negative effects on nontarget insects, such as parasitoid wasps and pollinators (162). Several studies reported that the OBP either showed high binding affinity to insecticides (154, 158) or the binding of OBP to floral volatile was significantly affected by insecticides (163). These studies implied that OBPs may contribute to olfaction based behavioral response to insecticides. In addition to synthetic pesticides, insect OBPs play roles in adaptation to biopesticides (e.g. essential oils) that are derived from natural materials, including plants, microorganisms, and other biological sources. For example, the *TCOBPC11* (*T. castaneum*) gene expression was induced in response to the essential oils of *Artemisa vulgaris* in the late instar larvae (26). Gene silencing of *TCOBPC11* by RNAi led to higher mortality in larvae compared with the control larvae treated with essential oils, suggesting that TCOBPC11 may play a role in resistance by sequestrating of plant essential oils and masking the toxic effects.

Host plant and pesticide adaptation might be linked due to chemical, evolutionary, and ecological evidence in detoxification and chemosensory pathways (22, 33, 34, 77, 164–166). It is possible that the capability associated with OBP-mediated pheromone or host plant adaptation in herbivorous insects has been co-opted for pesticide adaptation when they are exposed to pesticides. Most recently, research reported that insect OBPs can bind sex pheromone components, plant volatiles and pesticides (79, 153, 159). An OBP (AlepGOBP2) that was functionally characterized in the polyphagous insect *A. lepigone* showed high binding affinity to two conspecific sex pheromones ((Z)-7-dodecenyl acetate and (Z)-9-tetradecenyl acetate), two maize plant volatiles (Ocimene and (E)-β-Farnesene), and two organophosphate insecticides (chlorpyrifos and phoxim) (79). These results indicated AlepGOBP2 may facilitate recognition and adaptation to sex pheromones, plant volatiles, and insecticides all together.

In summary, current studies suggest that insect OBPs contribute to pesticide adaptation through sequestration and subsequent masking of the harmful effects of toxic compounds, or by acting as phase 0 transport proteins and shuttling toxic compounds across the cell membrane to phase I and/or phase II enzymes for further processing (27, 167–169). Whether this is accomplished solely by insect OBPs or through the assistance of other proteins, such as detoxification enzymes, remains to be elucidated.

## 4 Conclusion

While our understanding of insect OBPs was initially centered on olfaction, recent research conducted over the past decade has unveiled their involvement in diverse physiological processes, including communication, host location and adaptation, pesticide resistance, and reproduction. However, our comprehension of the molecular mechanisms governing OBP functions beyond olfaction remains limited due to their substantial diversity across various taxa. Recent advances in whole genomic sequences, RNA interference, gene editing, X-ray crystallography, and fluorescent competitive ligand binding assays, promise to enhance our understanding on the roles of insect OBPs towards communication and xenobiotic adaptation. This cutting-edge research will also contribute to unraveling the intricate and multifaceted mechanisms underpinning the evolutionary relationship between insects and their environment.

## Author contributions

JA: Methodology, Visualization, Writing – original draft, Data curation, Investigation, Software. TM: Investigation, Software, Visualization, Resources, Writing – review & editing. HW: Resources, Software, Visualization, Writing – review & editing, Data curation, Methodology. FZ: Methodology, Resources, Visualization, Writing – review & editing, Conceptualization, Funding acquisition, Project administration, Supervision, Validation, Writing – original draft.

## Supplementary material

Supplementary Table 1 Summary of odorant binding proteins (OBPs) used in the creation of the phylogenetic trees (**Figure 2**). Sequences without a complementary accession number were adapted from prior literature, where sequences were referenced but lacked an accession number.

## References

[1] Rihani K Ferveur JF Briand L. The 40-year mystery of insect odorant-binding proteins. Biomolecules (2021) 11(4):27. doi: 10.3390/biom11040509 PMC8067015 33808208

[2] Pelosi P Iovinella I Zhu J Wang GR Dani FR. Beyond chemoreception: diverse tasks of soluble olfactory proteins in insects. Biol Rev (2018) 93(1):184–200. doi: 10.1111/brv.12339 28480618

[3] Leal WS. Odorant reception in insects: roles of receptors, binding proteins, and degrading enzymes. Annu Rev Entomol (2013) 58(1):373–91. doi: 10.1146/annurev-ento-120811-153635 23020622

[4] Pelosi P Calvello M Ban LP. Diversity of odorant-binding proteins and chemosensory proteins in insects. Chem Senses (2005) 30:I291–i2. doi: 10.1093/chemse/bjh229 15738163

[5] Homberg U Christensen TA Hildebrand JG. Structure and function of the deutocerebrum in insects. Annu Rev Entomol (1989) 34:477–501. doi: 10.1146/annurev.en.34.010189.002401 2648971

[6] Baker TC Fadamiro HY Cosse AA. Moth uses fine tuning for odour resolution. Nature (1998) 393(6685):530. doi: 10.1038/31131

[7] Leal WS. Pheromone reception. In: Schulz S, editor. The chemistry of pheromones and other Semiochemicals II. Berlin, Heidelberg: Springer Berlin Heidelberg (2005). p. 1–36.

[8] de Bruyne M Baker TC. Odor detection in insects: volatile codes. J Chem Ecol (2008) 34(7):882–97. doi: 10.1007/s10886-008-9485-4 18535862

[9] Brito NF Moreira MF Melo ACA. A look inside odorant-binding proteins in insect chemoreception. J Insect Physiol (2016) 95:51–65. doi: 10.1016/j.jinsphys.2016.09.008 27639942

[10] Wu H Liu Y Shi X Zhang X Ye C Zhu KY. Transcriptome analysis of antennal cytochrome P450s and their transcriptional responses to plant and locust volatiles in *Locusta migratoria* . Int J Biol Macromol (2020) 149:741–53. doi: 10.1016/j.ijbiomac.2020.01.309 32018005

[11] Wei H Tan S Li Z Li J Moural TW Zhu F. Odorant degrading carboxylesterases modulate foraging and mating behaviors of *Grapholita molesta* . Chemosphere (2021) 270:128647. doi: 10.1016/j.chemosphere.2020.128647 33757271

[12] Koirala BKS Moural T Zhu F. Functional and structural diversity of insect glutathione S-transferases in xenobiotic adaptation. Int J Biol Sci (2022) 18(15):5713–23. doi: 10.7150/ijbs.77141 PMC9576527 36263171

[13] Cruse C Moural TW Zhu F. Dynamic roles of insect carboxyl/cholinesterases in chemical adaptation. Insects (2023) 14(2):194. doi: 10.3390/insects14020194 36835763 PMC9958613

[14] Kaissling KE. Olfactory perireceptor and receptor events in moths: a kinetic model. Chem Senses (2001) 26(2):125–50. doi: 10.1007/s00359-009-0461-4 11238244

[15] Kaissling KE. Olfactory perireceptor and receptor events in moths: a kinetic model revised. J Comp Physiol (2009) 195(10):895–922. doi: 10.1007/s00359-009-0461-4 19697043 PMC2749182

[16] Vogt RG Riddiford LM. Pheromone binding and inactivation by moth antennae. Nature (1981) 293(5828):161–3. doi: 10.1038/293161a0 18074618

[17] Gong Y Pace TCS Castillo C Bohne C O’Neill MA Plettner E. Ligand-interaction kinetics of the pheromone-binding protein from the gypsy moth, *L. dispar*: insights into the mechanism of binding and release. Chem Biol (2009) 16(2):162–72. doi: 10.1016/j.chembiol.2009.01.005 19246007

[18] Carey AF Carlson JR. Insect olfaction from model systems to disease control. Proc Natl Acad Sci U.S.A. (2011) 108(32):12987–95. doi: 10.1073/pnas.110347210 PMC3156210 21746926

[19] Clyne PJ Warr CG Freeman MR Lessing D Kim JH Carlson JR. A novel family of divergent seven-transmembrane proteins: candidate odorant receptors in *Drosophila* . Neuron (1999) 22(2):327–38. doi: 10.1016/s0896-6273(00)81093-4 10069338

[20] Pelosi P. Odorant-binding proteins. Crit Rev Biochem Mol Biol (1994) 29(3):199–228. doi: 10.3109/10409239409086801 8070277

[21] Bautista MAM Bhandary B Wijeratne AJ Michel AP Hoy CW Mittapalli O. Evidence for trade-offs in detoxification and chemosensation gene signatures in *Plutella xylostella* . Pest Manag Sci (2015) 71(3):423–32. doi: 10.1002/ps.3822 24796243

[22] Whiteman NK Pierce NE. Delicious poison: genetics of drosophila host plant preference. Trends Ecol Evol (2008) 23(9):473–8. doi: 10.1016/j.tree.2008.05.010 18657878

[23] Steinbrecht RA. Odorant-binding proteins: expression and function. Ann N Y. Acad Sci (1998) 855:323–32. doi: 10.1111/j.1749-6632.1998.tb10591.x 10049226

[24] Tricoire-Leignel H Thany SH Gadenne C Anton S. Pest insect olfaction in an insecticide-contaminated environment: info-disruption or hormesis effect. Front Physiol (2012) 3:58. doi: 10.3389/fphys.2012.00058 22457653 PMC3307139

[25] Lin X Jiang Y Zhang L Cai Y. Effects of insecticides chlorpyrifos, emamectin benzoate and fipronil on *Spodoptera litura* might be mediated by OBPs and CSPs. Bull Entomol Res (2018) 108(5):658–66. doi: 10.1017/S0007485317001195 29198202

[26] Zhang YC Gao SS Xue S Zhang KP Wang JS Li B. Odorant-binding proteins contribute to the defense of the red flour beetle, *Tribolium castaneum*, against essential oil of *Artemisia vulgaris* . Front Physiol (2020) 11:819. doi: 10.3389/fphys.2020.00819 32982763 PMC7488584

[27] Xiong WF Gao SS Lu YY Wei LT Mao JJ Xie J. Latrophilin participates in insecticide susceptibility through positively regulating CSP10 and partially compensated by OBPC01 in *Tribolium castaneum* . Pestic Biochem Phys (2019) 159:107–17. doi: 10.1016/j.pestbp.2019.06.005 31400772

[28] Matsuo T Sugaya S Yasukawa J Aigaki T Fuyama Y. Odorant-binding proteins OBP57d and OBP57e affect taste perception and host-plant preference in *Drosophila sechellia* . PloS Biol (2007) 5(5):985–96. doi: 10.1371/journal.pbio.0050118 PMC1854911 17456006

[29] Balabanidou V Kefi M Aivaliotis M Koidou V Girotti JR Mijailovsky SJ. Mosquitoes cloak their legs to resist insecticides. Proc Biol Sci (2019) 286(1907):20191091. doi: 10.1098/rspb.2019.1091 31311476 PMC6661348

[30] Sun JS Xiao S Carlson JR. The diverse small proteins called odorant-binding proteins. Open Biol (2018) 8(12):180208. doi: 10.1098/rsob.180208 30977439 PMC6303780

[31] Schoonhoven LM van Loon JJA Dicke M. Insect-plant biology. Oxford: Oxford University Press (2005).

[32] Irwin RE Cook D Richardson LL Manson JS Gardner DR. Secondary compounds in floral rewards of toxic rangeland plants: impacts on pollinators. J Agric Food Chem (2014) 62(30):7335–44. doi: 10.1021/jf500521w 24766254

[33] Alyokhin A Chen YH. Adaptation to toxic hosts as a factor in the evolution of insecticide resistance. Curr Opin Insect Sci (2017) 21:33–8. doi: 10.1016/j.cois.2017.04.006 28822486

[34] Despres L David JP Gallet C. The evolutionary ecology of insect resistance to plant chemicals. Trends Ecol Evol (2007) 22(6):298–307. doi: 10.1016/j.tree.2007.02.010 17324485

[35] Schoville SD Chen YH Andersson MN Benoit JB Bhandari A Bowsher JH. A model species for agricultural pest genomics: the genome of the Colorado potato beetle, *Leptinotarsa decemlineata* (Coleoptera: Chrysomelidae). Sci Rep (2018) 8:18. doi: 10.1038/s41598-018-20154-1 29386578 PMC5792627

[36] Zhou JJ. Odorant-binding proteins in insects. In: Litwack G, editor. Vitamins and hormones, vol. 83. Amsterdam, The Netherlands: Elsevier (2010). 10.1016/S0083-6729(10)83010-9 20831949

[37] Saitou N Nei M. The neighbor-joining method: a new method for reconstructing phylogenetic trees. Molec Biol Evol (1987) 4(4):406–25. doi: 10.1093/oxfordjournals.molbev.a040454 3447015

[38] Tamura K Stecher G Kumar S. MEGA11: molecular evolutionary genetics analysis version 11. Molec Biol Evol (2021) 38(7):3022–7. doi: 10.1093/molbev/msab120 PMC8233496 33892491

[39] Vieira FG Rozas J. Comparative genomics of the odorant-binding and chemosensory protein gene families across the arthropoda: origin and evolutionary history of the chemosensory system. Genome Biol Evol (2011) 3:476–90. doi: 10.1093/gbe/evr033 PMC3134979 21527792

[40] Mam B Karpe SD Sowdhamini R. Minus-C subfamily has diverged from classic odorant-binding proteins in honeybees. Apidologie (2023) 54(1):16. doi: 10.1007/s13592-022-00988-5

[41] Foret S Maleszka R. Function and evolution of a gene family encoding odorant binding-like proteins in a social insect, the honey bee (*Apis mellifera*). Genome Res (2006) 16(11):1404–13. doi: 10.1101/gr.5075706 PMC1626642 17065610

[42] Zhang B Zhang W Nie R-E Li W-Z Segraves KA Yang X-K. Comparative transcriptome analysis of chemosensory genes in two sister leaf beetles provides insights into chemosensory speciation. Insect Biochem Mol Biol (2016) 79:108–18. doi: 10.1016/j.ibmb.2016.11.001 27836740

[43] Wu Z Bin S He H Wang Z Li M Lin J. Differential expression analysis of chemoreception genes in the striped flea beetle *Phyllotreta striolata* using a transcriptomic approach. PloS One (2016) 11(4):e0153067. doi: 10.1371/journal.pone.0153067 27064483 PMC4827873

[44] McKenna DD Scully ED Pauchet Y Hoover K Kirsch R Geib SM. Genome of the Asian longhorned beetle (*Anoplophora glabripennis*), a globally significant invasive species, reveals key functional and evolutionary innovations at the beetle–plant interface. Genome Biol (2016) 17(1):227. doi: 10.1186/s13059-016-1088-8 27832824 PMC5105290

[45] Liu S Rao XJ Li MY Feng MF He MZ Li SG. Identification of candidate chemosensory genes in the antennal transcriptome of *Tenebrio molitor* (Coleoptera: tenebrionidae). Comp Biochem Physiol – D: Genom Proteom (2015) 13:44–51. doi: 10.1016/j.cbd.2015.01.004 25665775

[46] Li XM Zhu XY Wang ZQ Wang Y He P Chen G. Candidate chemosensory genes identified in *Colaphellus bowringi* by antennal transcriptome analysis. BMC Genom (2015) 16:16. doi: 10.1186/s12864-015-2236-3 PMC4667470 26626891

[47] Andersson MN Grosse-Wilde E Keeling CI Bengtsson JM Yuen MMS Li M. Antennal transcriptome analysis of the chemosensory gene families in the tree killing bark beetles, *Ips typographus* and *Dendroctonus ponderosae* (Coleoptera: curculionidae: scolytinae). BMC Genom (2013) 14:16. doi: 10.1186/1471-2164-14-198 PMC3610139 23517120

[48] Xu PX Zwiebel LJ Smith DP. Identification of a distinct family of genes encoding atypical odorant-binding proteins in the malaria vector mosquito, *Anopheles gambiae* . Insect Mol Biol (2003) 12(6):549–60. doi: 10.1046/j.1365-2583.2003.00440.x 14986916

[49] Jiang X Krieger J Breer H Pregitzer P. Distinct subfamilies of odorant binding proteins in locust (Orthoptera, acrididae): molecular evolution, structural variation, and sensilla-specific expression. Front Physiol (2017) 8:734. doi: 10.3389/fphys.2017.00734 29018357 PMC5623057

[50] Guo W Ren D Zhao L Jiang F Song J Wang X. Identification of odorant-binding proteins (OBPs) and functional analysis of phase-related OBPs in the migratory locust. Front Physiol (2018) 9:984. doi: 10.3389/fphys.2018.00984 30079035 PMC6062766

[51] Vieira FG Foret S He XL Rozas J Field LM Zhou JJ. Unique features of odorant-binding proteins of the parasitoid wasp *Nasonia vitripennis* revealed by genome annotation and comparative analyses. PloS One (2012) 7(8):11. doi: 10.1371/journal.pone.0043034 PMC3428353 22952629

[52] Leite NR Krogh R Xu W Ishida Y Iulek J Leal WS. Structure of an odorant-binding protein from the mosquito *Aedes aegypti* suggests a binding pocket covered by a pH-sensitive “lid”. PloS One (2009) 4(11):7. doi: 10.1371/journal.pone.0008006 PMC2778553 19956631

[53] Pelosi P Maida R. Odorant-binding proteins in insects. Comp Biochem Physiol - B Biochem Mol Biol (1995) 111(3):503–14. doi: 10.1016/0305-0491(95)00019-5 7613772

[54] Li N Sun X Wang MQ. Expression pattern and ligand-binding properties of odorant-binding protein 13 from *Monochamus alternatus* hope. J Appl Entomol (2017) 141(9):751–7. doi: 10.1111/jen.12396

[55] Pelosi P Iovinella I Felicioli A Dani FR. Soluble proteins of chemical communication: an overview across arthropods. Front Physiol (2014) 5:320. doi: 10.3389/fphys.2014.00320 25221516 PMC4145409

[56] Laughlin JD Ha TS Jones DNM Smith DP. Activation of pheromone-sensitive neurons is mediated by conformational activation of pheromone-binding protein. Cell (2008) 133(7):1255–65. doi: 10.1016/j.cell.2008.04.046 PMC4397981 18585358

[57] Zhu J Renzone G Arena S Dani FR Paulsen H Knoll W. The odorant-binding proteins of the spider mite *Tetranychus urticae* . Int J Mol Sci (2021) 22(13):6828. doi: 10.3390/ijms22136828 PMC8269058 34202019

[58] Campanini EB de Brito RA. Molecular evolution of odorant-binding proteins gene family in two closely related *Anastrepha* fruit flies. BMC Evol Biol (2016) 16:16. doi: 10.1186/s12862-016-0775-0 27716035 PMC5054612

[59] Tsitsanou KE Drakou CE Thireou T Gruber AV Kythreoti G Azem A. Crystal and solution studies of the “plus-C” odorant-binding protein 48 from *Anopheles gambiae* control of binding specificity through three-dimensional domain swapping. J Biol Chem (2013) 288(46):33427–38. doi: 10.1074/jbc.M113.505289 PMC3829188 24097978

[60] Lagarde A Spinelli S Tegoni M He XL Field L Zhou JJ. The crystal structure of odorant binding protein 7 from *Anopheles gambiae* exhibits an outstanding adaptability of its binding site. J Mol Biol (2011) 414(3):401–12. doi: 10.1016/j.jmb.2011.10.005 22019737

[61] Riviere S Lartigue A Quennedey B Campanacci V Farine JP Tegoni M. A pheromone-binding protein from the cockroach *Leucophaea maderae*: cloning, expression and pheromone binding. Biochem J (2003) 371:573–9. doi: 10.1042/BJ20021877 PMC1223297 12529170

[62] Sandler BH Nikonova L Leal WS Clardy J. Sexual attraction in the silkworm moth: structure of the pheromone-binding-protein-bombykol complex. Chem Biol (2000) 7(2):143–51. doi: 10.1016/s1074-5521(00)00078-8 10662696

[63] Leal WS Chen AM Ishida Y Chiang VP Erickson ML Morgan TI. Kinetics and molecular properties of pheromone binding and release. Proc Natl Acad Sci USA (2005) 102(15):5386–91. doi: 10.1073/pnas.0501447102 PMC555038 15784736

[64] Horst R Damberger F Luginbuhl P Guntert P Peng G Nikonova L. NMR structure reveals intramolecular regulation mechanism for pheromone binding and release. Proc Natl Acad Sci USA (2001) 98(25):14374–9. doi: 10.1073/pnas.251532998 PMC64689 11724947

[65] Wojtasek H Leal WS. Conformational change in the pheromone-binding protein from *Bombyx mori* induced by pH and by interaction with membranes. J Biol Chem (1999) 274(43):30950–6. doi: 10.1074/jbc.274.43.30950 10521490

[66] Xu XZ Xu W Rayo J Ishida Y Leal WS Ames JB. NMR structure of navel orangeworm moth pheromone-binding protein (AtraPBP1): implications for pH-sensitive pheromone detection. Biochemistry (2010) 49(7):1469–76. doi: 10.1021/bi9020132 PMC2822879 20088570

[67] Zubkov S Gronenborn AM Byeon IJL Mohanty S. Structural consequences of the pH-induced conformational switch in A. polyphemus pheromone-binding protein: mechanisms of ligand release. J Mol Biol (2005) 354(5):1081–90. doi: 10.1016/j.jmb.2005.10.015 16289114

[68] Li DZ Yu GQ Yi SC Zhang YN Kong DX Wang MQ. Structure-based analysis of the ligand-binding mechanism for DhelOBP21, a C-minus odorant binding protein, from *Dastarcus helophoroides* (Fairmaire; Coleoptera: bothrideridae). Int J Biol Sci (2015) 11(11):1281–95. doi: 10.7150/ijbs.12528 PMC4582152 26435694

[69] Wei HS Duan HX Li KB Zhang S Wei ZJ Yin J. The mechanism underlying OBP heterodimer formation and the recognition of odors in *Holotrichia oblita* faldermann. Int J Biol Macromol (2020) 152:957–68. doi: 10.1016/j.ijbiomac.2019.10.182 31778700

[70] Tsitsanou KE Thireou T Drakou CE Koussis K Keramioti MV Leonidas DD. *Anopheles gambiae* odorant binding protein crystal complex with the synthetic repellent DEET: implications for structure-based design of novel mosquito repellents. Cell Mol Life Sci (2012) 69(2):283–97. doi: 10.1007/s00018-011-0745-z PMC11114729 21671117

[71] Lagarde A Spinelli S Qiao HL Tegoni M Pelosi P Cambillau C. Crystal structure of a novel type of odorant-binding protein from *Anopheles gambiae*, belonging to the C-plus class. Biochem J (2011) 437:423–30. doi: 10.1042/BJ20110522 21561433

[72] Mao Y Xu XZ Xu W Ishida Y Leal WS Ames JB. Crystal and solution structures of an odorant-binding protein from the southern house mosquito complexed with an oviposition pheromone. Proc Natl Acad Sci USA (2010) 107(44):19102–7. doi: 10.1073/pnas.101227410 PMC2973904 20956299

[73] Kruse SW Zhao R Smith DP Jones DNM. Structure of a specific alcohol-binding site defined by the odorant binding protein LUSH from *Drosophila melanogaster* . Nat Struct Biol (2003) 10(9):694–700. doi: 10.1038/nsb960 12881720 PMC4397894

[74] Pelosi P Zhu J Knoll W. Odorant-binding proteins as sensing elements for odour monitoring. Sensors (2018) 18(10):3248. doi: 10.3390/s18103248 PMC6210013 30262737

[75] Joseph RM Carlson JR. *Drosophila* chemoreceptors: a molecular interface between the chemical world and the brain. Trends Genet (2015) 31(12):683–95. doi: 10.1016/j.tig.2015.09.005 PMC4674303 26477743

[76] Zhu F Cui Y Walsh DB Lavine LC. Application of RNAi towards insecticide resistance management. In: Chandrasekar R Tyagi BK Gui Z Reeck GR, editors. Short Views on Insect Biochemistry and Molecular Biology. Manhattan, USA: Academic Publisher (2014). p. 595–619.

[77] Schuler MA. The role of cytochrome P450 monooxygenases in plant-insect interactions. Plant Physiol (1996) 112(4):1411–9. doi: 10.1104/pp.112.4.1411 PMC158072 8972591

[78] Wheat CW Vogel H Wittstock U Braby MF Underwood D Mitchell-Olds T. The genetic basis of a plant-insect coevolutionary key innovation. Proc Natl Acad Sci USA (2007) 104(51):20427–31. doi: 10.1073/pnas.0706229104 PMC2154447 18077380

[79] Zhang XQ Yan Q Li LL Xu JW Mang DZ Wang XL. Different binding properties of two general-odorant binding proteins in *Athetis lepigone* with sex pheromones, host plant volatiles and insecticides. Pestic Biochem Phys (2020) 164:173–82. doi: 10.1016/j.pestbp.2020.01.012 32284124

[80] Zhao H Peng Z Huang L Zhao S Liu M. Expression profile and ligand screening of a putative odorant-binding protein, AcerOBP6, from the asian honeybee. Insects (2021) 12(11):955. doi: 10.3390/insects12110955 PMC8622152 34821756

[81] Liu NY Yang F Yang K He P Niu XH Xu W. Two subclasses of odorant-binding proteins in *Spodoptera exigua* display structural conservation and functional divergence. Insect Mol Biol (2015) 24(2):167–82. doi: 10.1111/imb.12143 25345813

[82] Dani FR Iovinella I Felicioli A Niccolini A Calvello MA Carucci MG. Mapping the expression of soluble olfactory proteins in the honeybee. J Proteome Res (2010) 9(4):1822–33. 10.1021/pr900969k 20155982

[83] Dani FR Michelucci E Francese S Mastrobuoni G Cappellozza S La Marca G. Odorant-binding proteins and chemosensory proteins in pheromone detection and release in the silkmoth *Bombyx mori* . Chem Senses (2011) 36(4):335–44. doi: 10.1093/chemse/bjq137 21220518

[84] Iovinella I Dani FR Niccolini A Sagona S Michelucci E Gazzano A. Differential expression of odorant-binding proteins in the mandibular glands of the honey bee according to caste and age. J Proteome Res (2011) 10:984(8). doi: 10.3389/fphys.2018.00984 21707107

[85] Yang HB Dong JF Sun YL Hu ZJ Lv QH Li DX. Antennal transcriptome analysis and expression profiles of putative chemosensory soluble proteins in *Histia rhodope* Cramer (Lepidoptera: Zygaenidae). Comp Biochem Physiol Part D Genomics Proteomics (2020) 33:13. doi: 10.1016/j.cbd.2020.100654 31954363

[86] Deisig N Dupuy F Anton S Renou M. Responses to pheromones in a complex odor world: sensory processing and behavior. Insects (2014) 5(2):399–422. doi: 10.3390/insects5020399 26462691 PMC4592597

[87] Renou M. Pheromones and general odor perception in insects. In: Mucignat-Caretta C, editor. Neurobiology of Chemical Communication, vol. chapter 2 . Boca Raton (FL: CRC Press/Taylor & Francis (2014). 24830044

[88] Wyatt TD. Pheromones and animal behavior: chemical signals and signatures. Cambridge, University Printing House Shaftesbury Road, United Kingdom: Cambridge University Press (2014). doi: 10.1017/CBO9781139030748

[89] Zhang J Walker WB Wang G. Pheromone reception in moths: from molecules to behaviors. Prog Mol Biol Transl Sci (2015) 130:109–28. doi: 10.1016/bs.pmbts.2014.11.005 25623339

[90] Zhu G-H Xu J Cui Z Dong X-T Ye Z-F Niu D-J. Functional characterization of SlitPBP3 in *Spodoptera litura* by CRISPR/Cas9 mediated genome editing. Insect Biochem Molec Biol (2016) 75:619816. doi: 10.3389/fphys.2021.619816 27192033

[91] Ma S Li LL Yao WC Yin MZ Li JQ Xu JW. Two odorant-binding proteins involved in the recognition of sex pheromones in *Spodoptera litura* larvae. J Agric Food Chem (2022) 70(39):12372–82. doi: 10.1021/acs.jafc.2c04335 36129378

[92] Sun M Liu Y Wang G. Expression patterns and binding properties of three pheromone binding proteins in the diamondback moth. Plutella xyllotella. J Insect Physiol (2013) 59(1):46–55. doi: 10.1016/j.jinsphys.2012.10.020 23147025

[93] Qin JH Wang CQ Li KB Cao YZ Peng Y Feng HL. Molecular characterization of sex pheromone binding proteins from *Holotrichia oblita* (Coleoptera: scarabaeida). Int J Biol Macromol (2021) 193(Pt A):8–18. doi: 10.1016/j.ijbiomac.2021.10.059 34673107

[94] Zhang YN Zhang XQ Zhang XC Xu JW Li LL Zhu XY. Key amino acid residues influencing binding affinities of pheromone-binding protein from *Athetis lepigone* to two sex pheromones. J Agric Food Chem (2020) 68(22):6092–103. doi: 10.1021/acs.jafc.0c01572 32392414

[95] Grosse-Wilde E Svatos A Krieger J. A pheromone-binding protein mediates the bombykol-induced activation of a pheromone receptor in *vitro* . Chem Senses (2006) 31(6):547–55. doi: 10.1093/chemse/bjj059 16679489

[96] Syed Z Ishida Y Taylor K Kimbrell DA Leal WS. Pheromone reception in fruit flies expressing a moth’s odorant receptor. Proc Natl Acad Sci USA (2006) 103(44):16538–43. doi: 10.1073/pnas.0607874103 PMC1621046 17060610

[97] Forstner M Gohl T Breer H Krieger J. Candidate pheromone binding proteins of the silkmoth *Bombyx mori* . Invert Nuerosci (2006) 6(4):177–87. doi: 10.1007/s10158-006-0032-0 17082917

[98] Michel E Damberger FF Ishida Y Fiorito F Lee D Leal WS. Dynamic conformational equilibria in the physiological function of the *Bombyx mori* pheromone-binding protein. J Mol Biol (2011) 408(5):922–31. doi: 10.1016/j.jmb.2011.03.008 21396939

[99] Damberger FF Ishida Y Leal WS Wüthrich K. Structural basis of ligand binding and release in insect pheromone-binding proteins: NMR structure of *Antheraea polyphemus* PBP1 at pH 4.5. J Mol Biol (2007) 373(4):811–9. doi: 10.1016/j.jmb.2007.07.078 17884092

[100] Xu W Xu X Leal WS Ames JB. Extrusion of the C-terminal helix in navel orangeworm moth pheromone-binding protein (AtraPBP1) controls pheromone binding. Biochem Biophys Res Commun (2011) 404(1):335–8. doi: 10.1016/j.bbrc.2010.11.119 PMC3019287 21130734

[101] Xu PX Atkinson R Jones DNM Smith DP. *Drosophila* OBP LUSH is required for activity of pheromone-sensitive neurons. Neuron (2005) 45(2):193–200. doi: 10.1016/j.neuron.2004.12.031 15664171

[102] Gomez-Diaz C Reina JH Cambillau C Benton R. Ligands for pheromone-sensing neurons are not conformationally activated odorant binding proteins. PloS Biol (2013) 11(4):e1001546. doi: 10.1371/journal.pbio.1001546 23637570 PMC3640100

[103] Swarup S Williams TI Anholt RRH. Functional dissection of odorant binding protein genes in *Drosophila melanogaster* . Genes Brain Behav (2011) 10(6):648–57. doi: 10.1111/j.1601-183X.2011.00704.x PMC3150612 21605338

[104] Shorter JR Dembeck LM Everett LJ Morozova TV Arya GH Turlapati L. Obp56h modulates mating behavior in *Drosophila melanogaster* . G3-Genes Genom Genet (2016) 6(10):3335–42. doi: 10.1534/g3.116.034595 PMC5068952 27558663

[105] McAfee A Chapman A Iovinella I Gallagher-Kurtzke Y Collins TF Higo H. A death pheromone, oleic acid, triggers hygienic behavior in honey bees (*Apis mellifera* L.). Sci Rep (2018) 8(1):5719. doi: 10.1038/s41598-018-24054-2 29632403 PMC5890279

[106] Guarna MM Melathopoulos AP Huxter E Iovinella I Parker R Stoynov N. A search for protein biomarkers links olfactory signal transduction to social immunity. BMC Genom (2015) 16(1):63. doi: 10.1186/s12864-014-1193-6 PMC4342888 25757461

[107] Weng C Fu Y Jiang H Zhuang S Li H. Binding interaction between a queen pheromone component HOB and pheromone binding protein ASP1 of *Apis cerana* . Int J Biol Macromol (2015) 72:430–6. doi: 10.1016/j.ijbiomac.2014.08.046 25195542

[108] Pesenti ME Spinelli S Bezirard V Briand L Pernollet JC Tegoni M. Structural basis of the honey bee PBP pheromone and pH-induced conformational change. J Mol Biol (2008) 380(1):158–69. doi: 10.1016/j.jmb.2008.04.048 18508083

[109] Vandermoten S Francis F Haubruge E Leal WS. Conserved odorant-binding proteins from aphids and eavesdropping predators. PloS One (2011) 6(8):e23608. doi: 10.1371/journal.pone.0023608 21912599 PMC3160308

[110] Sun YF De Biasio F Qiao HL Iovinella I Yang SX Ling Y. Two odorant-binding proteins mediate the behavioural response of aphids to the alarm pheromone (E)-ß-farnesene and structural analogues. PloS One (2012) 7(3):e32759. doi: 10.1371/journal.pone.0032759 22427877 PMC3299684

[111] Zhong T Yin J Deng S Li K Cao Y. Fluorescence competition assay for the assessment of green leaf volatiles and trans-β-farnesene bound to three odorant-binding proteins in the wheat aphid *Sitobion avenae* (Fabricius). J Insect Physiol (2012) 58(6):771–81. doi: 10.1016/j.jinsphys.2012.01.011 22306433

[112] Li ZQ Zhang S Cai XM Luo JY Dong SL Cui JJ. Distinct binding affinities of odorant-binding proteins from the natural predator *Chrysoperla sinica* suggest different strategies to hunt prey. J Insect Physiol (2018) 111:25–31. doi: 10.1016/j.jinsphys.2018.10.004 30336148

[113] Qiao H Tuccori E He X Gazzano A Field L Zhou J-J. Discrimination of alarm pheromone (E)-β-farnesene by aphid odorant-binding proteins. Insect Biochem Mol Biol (2009) 39(5):414–9. doi: 10.1016/j.ibmb.2009.03.004 19328854

[114] Fan J Xue W Duan H Jiang X Zhang Y Yu W. Identification of an intraspecific alarm pheromone and two conserved odorant-binding proteins associated with (E)-β-farnesene perception in aphid *Rhopalosiphum padi* . J Insect Physiol (2017) 101:151–60. doi: 10.1016/j.jinsphys.2017.07.014 28778653

[115] Zhang R Wang B Grossi G Falabella P Liu Y Yan S. Molecular basis of alarm pheromone detection in aphids. Curr Biol (2017) 27(1):55–61. doi: 10.1016/j.cub.2016.10.013 27916525

[116] Tang H Xie J Liu J Khashaveh A Liu X Yi C. Odorant-binding protein HvarOBP5 in ladybird *Hippodamia variegata* regulates the perception of semiochemicals from preys and habitat plants. J Agric Food Chem (2023) 71(2):1067–76. doi: 10.1021/acs.jafc.2c07355 36598383

[117] Qu C Yang ZK Wang S Zhao HP Li FQ Yang XL. Binding affinity characterization of four antennae-enriched odorant-binding proteins from *Harmonia axyridis* (Coleoptera: coccinellidae). Front Physiol (2022) 13:829766. doi: 10.3389/fphys.2022.829766 35350682 PMC8957989

[118] Northey T Venthur H De Biasio F Chauviac FX Cole A Ribeiro KAJ. Crystal structures and binding dynamics of odorant-binding protein 3 from two aphid species Megoura viciae and *Nasonovia ribisnigri* . Sci Rep (2016) 6:24739. doi: 10.1038/srep24739 27102935 PMC4840437

[119] Li ZQ Zhang S Cai XM Luo JY Dong SL Cui JJ. Three odorant binding proteins may regulate the behavioural response of *Chrysopa pallens* to plant volatiles and the aphid alarm pheromone (E)-β-farnesene. Insect Mol Biol (2017) 26(3):255–65. doi: 10.7717/peerj.6576 28247518

[120] Song LM Jiang X Wang XM Li JD Zhu F Tu XB. Male tarsi specific odorant-binding proteins in the diving beetle *Cybister japonicus* sharp. Sci Rep (2016) 6:10. doi: 10.1038/srep31848 27545810 PMC4992826

[121] Findlay GD Yi XH MacCoss MJ Swanson WJ. Proteomics reveals novel *Drosophila* seminal fluid proteins transferred at mating. PloS Biol (2008) 6(7):1417–26. doi: 10.1371/journal.pbio.0060178 PMC2486302 18666829

[122] Li S Picimbon JF Ji SD Kan YC Qiao CL Zhou JJ. Multiple functions of an odorant-binding protein in the mosquito *Aedes aegypti* . Biochem Biophys Res Commun (2008) 372(3):464–8. doi: 10.1016/j.bbrc.2008.05.064 18502197

[123] Takemori N Yamamoto MT. Proteome mapping of the *Drosophila melanogaster* male reproductive system. Proteomics (2009) 9(9):2484–93. doi: 10.1002/pmic.200800795 19343724

[124] Baer B Zareie R Paynter E Poland V Millar AH. Seminal fluid proteins differ in abundance between genetic lineages of honeybees. J Proteomics (2012) 75(18):5646–53. doi: 10.1016/j.jprot.2012.08.002 22981951

[125] Sun YL Huang LQ Pelosi P Wang CZ. Expression in antennae and reproductive organs suggests a dual role of an odorant-binding protein in two sibling *Helicoverpa* species. PloS One (2012) 7(1):11. doi: 10.1371/journal.pone.0030040 PMC3264552 22291900

[126] Xu JJ Baulding J Palli SR. Proteomics of *Tribolium castaneum* seminal fluid proteins: identification of an angiotensin-converting enzyme as a key player in regulation of reproduction. J Proteomics (2013) 78:83–93. doi: 10.1016/j.jprot.2012.11.011 23195916

[127] Dorus S Busby SA Gerike U Shabanowitz J Hunt DF Karr TL. Genomic and functional evolution of the *Drosophila melanogaster* sperm proteome. Nat Genet (2006) 38(12):1440–5. doi: 10.1038/ng1915 17099714

[128] Visser JH. Host-plant finding by insects - orientation, sensory input and search patterns. J Insect Physiol (1988) 34(3):259–68. doi: 10.1016/0022-1910(88)90056-X

[129] Bruce TJA Wadhams LJ Woodcock CM. Insect host location: a volatile situation. Trends Plant Sci (2005) 10(6):269–74. doi: 10.1016/j.tplants.2005.04.003 15949760

[130] Bernays EA Chapman RF. Host-plant selection by phytophagous insects. Berlin/Heidelberg, Germany: Springer Science & Business Media (2007). doi: 10.1007/b102508

[131] Li J Zhang L. Electroantennographic activity of 21 aliphatic compounds that bind well to a locust odorant-binding protein. Arch Insect Biochem Physiol (2022) 110(3):e21911. doi: 10.1002/arch.21911 35599375

[132] Yue Y Ma C Zhang Y Chen H-S Guo J-Y Liu T-H. Characterization and functional analysis of OcomOBP7 in *Ophraella communa* Lesage. Insects (2023) 14(2):190. doi: 10.3390/insects14020190 36835759 PMC9967674

[133] Zhu J Wang F Zhang Y Yang Y Hua D. Odorant-binding protein 10 From *Bradysia odoriphaga* (Diptera: sciaridae) binds volatile host plant compounds. J Insect Sci (2023) 23(1):7. doi: 10.1093/jisesa/iead004 PMC9894006 36729094

[134] Blackwell A Johnson SN. Electrophysiological investigation of larval water and potential oviposition chemo-attractants for *Anopheles gambiae* s.s. Ann Trop Med Parasitol (2000) 94(4):389–98. doi: 10.1080/00034983.2000.11813554 10945049

[135] Meijerink J Braks MAH Brack AA Adam W Dekker T Posthumus MA. Identification of olfactory stimulants for *Anopheles gambiae* from human sweat samples. J Chem Ecol (2000) 26(6):1367–82. doi: 10.1023/A:1005475422978

[136] Biessmann H Andronopoulou E Biessmann MR Douris V Dimitratos SD Eliopoulos E. The *Anopheles gambiae* odorant binding protein 1 (AgamOBP1) mediates indole recognition in the antennae of female mosquitoes. PloS One (2010) 5(3):e9471. doi: 10.1371/journal.pone.0009471 20208991 PMC2830424

[137] Legal L Chappe B Jallon JM. Molecular basis of *Morinda citrifolia* (L.): toxicity on drosophila. J Chem Ecol (1994) 20(8):1931–43. doi: 10.1007/BF02066234 24242720

[138] Harada E Haba D Aigaki T Matsuo T. Behavioral analyses of mutants for two odorant-binding protein genes, OBP57d and OBP57e, in *Drosophila melanogaster* . Genes Genet Syst (2008) 83(3):257–64. doi: 10.1266/ggs.83.257 18670137

[139] Liu H Wang C Qiu CL Shi JH Sun Z Hu XJ. A salivary odorant-binding protein mediates *Nilaparvata lugens* feeding and host plant phytohormone suppression. Int J Molec Sci (2021) 22(9):15. doi: 10.3390/ijms22094988 PMC8125829 34066665

[140] Yi SC Wu YH Yang RN Li DZ Abdelnabby H Wang MQ. A highly expressed antennae odorant-binding protein involved in recognition of herbivore-induced plant volatiles in *Dastarcus helophoroides* . Int J Molec Sci (2023) 24(4):19. doi: 10.3390/ijms24043464 PMC9962305 36834874

[141] Baldwin IT. Plant volatiles. Curr Biol (2010) 20(9):R392–7. doi: 10.1016/j.cub.2010.02.052 20462477

[142] Li P Zhu J Qin Y. Enhanced attraction of *Plutella xylostella* (Lepidoptera: plutellidae) to pheromone-baited traps with the addition of green leaf volatiles. J Econ Entomol (2012) 105(4):1149–56. doi: 10.1603/ec11109 22928292

[143] Xu H Turlings TCJ. Plant volatiles as mate-finding cues for insects. Trends Plant Sci (2018) 23(2):100–11. doi: 10.1016/j.tplants.2017.11.004 29229187

[144] Liu NY Yang K Liu Y Xu W Anderson A Dong SL. Two general-odorant binding proteins in *Spodoptera litura* are differentially tuned to sex pheromones and plant odorants. Comp Biochem Physiol Part A Mol Integr Physiol (2015) 180:23–31. doi: 10.1016/j.cbpa.2014.11.005 25460831

[145] Sun X Zhao ZF Zeng FF Zhang A Lu ZX Wang MQ. Functional characterization of a pheromone-binding protein from rice leaffolder *Cnaphalocrocis medinalis* in detecting pheromones and host plant volatiles. Bull Entomol Res (2016) 106(6):781–9. doi: 10.1017/S0007485316000560 27385278

[146] Song XM Zhang LY Fu XB Wu F Tan J Li HL. Various bee pheromones binding affinity, exclusive chemosensillar localization, and key amino acid sites reveal the distinctive characteristics of odorant-binding protein 11 in the eastern honey bee, *Apis cerana* . Front Physiol (2018) 9:422. doi: 10.3389/fphys.2018.00422 29740337 PMC5924804

[147] Sun L Wang Q Zhang Y Tu X Yan Y Wang Q. The sensilla trichodea-biased EoblPBP1 binds sex pheromones and green leaf volatiles in *Ectropis obliqua* Prout, a geometrid moth pest that uses type-II sex pheromones. J Insect Physiol (2019) 116:17–24. doi: 10.1016/j.jinsphys.2019.04.005 31009623

[148] Wang Q Wang Q Li H Sun L Zhang D Zhang Y. Sensilla localization and sex pheromone recognition of odorant binding protein OBP4 in the mirid plant bug *Adelphocoris lineolatus* (Goeze). J Insect Physiol (2020) 121:104012. doi: 10.1016/j.jinsphys.2020.104012 31911184

[149] Li LL Huang JR Xu JW Yao WC Yang HH Shao L. Ligand-binding properties of odorant-binding protein 6 in *Athetis lepigone* to sex pheromones and maize volatiles. Pest Manag Sci (2022) 78(1):52–62. doi: 10.3390/insects13121145 34418275

[150] Li J Yin J Yan J Zhang M Chen R Li S. Expression and functional analysis of an odorant binding protein PopeOBP16 from *Phthorimaea operculella* (Zeller). Int J Biol Macromol (2023) 242:124939. doi: 10.1016/j.ijbiomac.2023.124939 37207749

[151] Li G Chen X Li B Zhang G Li Y Wu J. Binding properties of general odorant binding proteins from the oriental fruit moth, *Grapholita molesta* (Busck) (Lepidoptera: tortricidae). PloS One (2016) 11(5):e0155096. doi: 10.1371/journal.pone.0155096 27152703 PMC4859520

[152] Du Y Xu K Zhao H Jiang Y Li H. Identification and functional characterization of AcerOBP15 from *Apis cerana cerana* (Hymenoptera: apidae). Apidologie (2021) 52(3):668–83. doi: 10.1007/s13592-021-00854-w

[153] Yin NN Yang AJ Wu C Xiao HY Guo YR Liu NY. Genome-wide analysis of odorant-binding proteins in *Papilio xuthus* with focus on the perception of two PxutGOBPs to host odorants and insecticides. J Agric Food Chem (2022) 70(35):10747–61. doi: 10.1021/acs.jafc.2c03396 36002911

[154] Qiu YL Wu F Zhang L Jiang HQ Chen JT Pan YJ. A sublethal dose of neonicotinoid imidacloprid precisely sensed and detoxified by a C-minus odorant-binding protein 17 highly expressed in the legs of *Apis cerana* . Sci Total Environ (2023) 885:163762. doi: 10.1016/j.scitotenv.2023.163762 37146819

[155] Zhang YN Xu JW Zhang XC Zhang XQ Li LL Yuan XH. Organophosphorus insecticide interacts with the pheromone-binding proteins of *Athetis lepigone*: implication for olfactory dysfunction. J Hazard Mater (2020) 397:11. doi: 10.1016/j.jhazmat.2020.122777 32388456

[156] Sun ZX Wang RM Du YF Gao BY Gui FR Lu K. Olfactory perception of herbicide butachlor by GOBP2 elicits ecdysone biosynthesis and detoxification enzyme responsible for chlorpyrifos tolerance in *Spodoptera litura* . Environ pollut (2021) 285:10. doi: 10.1016/j.envpol.2021.117409 34049133

[157] Zhang JJ Mao KK Ren ZJ Jin RH Zhang YH Cai TW. Odorant binding protein 3 is associated with nitenpyram and sulfoxaflor resistance in *Nilaparvata lugens* . Int J Biol Macromol (2022) 209:1352–8. doi: 10.1016/j.ijbiomac.2022.04.100 35460755

[158] Li YJ Hong TL Chen HC Gu FM Liu ZX You S. Odorant-binding protein 6 contributes high binding affinity to insecticides in a parasitic wasp *Meteorus pulchricornis* (Hymenoptera: braconidae). J Agric Food Chem (2023) 71(11):4498–509. doi: 10.1021/acs.jafc.2c08390 36883889

[159] Wu C Yin N Guo Y Wang Z Liu N. Two antenna-enriched odorant binding proteins in *Dioryctria abietella* tuned to general odorants and insecticides. Insects (2022) 13(12):1145. doi: 10.3390/insects13121145 36555056 PMC9781003

[160] Zhu F Gujar H Gordon JR Haynes KF Potter MF Palli SR. Bed bugs evolved unique adaptive strategy to resist pyrethroid insecticides. Sci Rep (2013) 3:1456. doi: 10.1038/srep01456 23492626 PMC3596983

[161] Liu XQ Jiang HB Liu Y Fan JY Ma YJ Yuan CY. Odorant binding protein 2 reduces imidacloprid susceptibility of *Diaphorina citri* . Pestic Biochem Physiol (2020) 168:7. doi: 10.1016/j.pestbp.2020.104642 32711775

[162] Desneux N Decourtye A Delpuech JM. The sublethal effects of pesticides on beneficial arthropods. Annu Rev Entomol (2007) 52:81–106. doi: 10.1146/annurev.ento.52.110405.091440 16842032

[163] Li H Wu F Zhao L Tan J Jiang H Hu F. Neonicotinoid insecticide interact with honeybee odorant-binding protein: implication for olfactory dysfunction. Int J Biol Macromol (2015) 81:624–30. doi: 10.1016/j.ijbiomac.2015.08.055 26318218

[164] Zhu F Moural TW Nelson DR Palli SR. A specialist herbivore pest adaptation to xenobiotics through up-regulation of multiple Cytochrome P450s. Sci Rep (2016) 6:20421. doi: 10.1038/srep20421 26861263 PMC4748221

[165] Zhu F Moural TW Shah K Palli SR. Integrated analysis of cytochrome P450 gene superfamily in the red flour beetle, *Tribolium castaneum* . BMC Genom (2013) 14:174. doi: 10.1186/1471-2164-14-174 PMC3682917 23497158

[166] Dermauw W Wybouw N Rombauts S Menten B Vontas J Grbic M. A link between host plant adaptation and pesticide resistance in the polyphagous spider mite *Tetranychus urticae* . Proc Natl Acad Sci USA (2013) 110(2):E113–22. doi: 10.1073/pnas.1213214110 PMC3545796 23248300

[167] Amezian D Nauen R Le Goff G. Transcriptional regulation of xenobiotic detoxification genes in insects - an overview. Pestic Biochem Physiol (2021) 174:104822. doi: 10.1016/j.pestbp.2021.104822 33838715

[168] Chen XH Xiong WF Li CJ Gao SS Song XW Wu W. Comparative RNA-sequencing profiling reveals novel Delta-class glutathione S-transferases relative genes expression patterns in *Tribolium castaneum* . Gene (2016) 593(1):13–20. doi: 10.1016/j.gene.2016.08.013 27511373

[169] Gao SS Xiong WF Wei LT Liu JJ Liu X Xie J. Transcriptome profiling analysis reveals the role of latrophilin in controlling development, reproduction and insecticide susceptibility in *Tribolium castaneum* . Genetica (2018) 146(3):287–302. doi: 10.1007/s10709-018-0020-4 29797154

