# Supplementary material for: Roles of insect odorant binding proteins in communication and xenobiotic adaptation
Source: Front Insect Sci. 2023 Oct 6;3:1274197. doi: 10.3389/finsc.2023.1274197 (PMC10926425; doi:10.3389/finsc.2023.1274197)
Supplement: Supplementary Table 1 — Summary of odorant binding proteins (OBPs) used in the creation of the phylogenetic trees ( Figure 2 ). Sequences without a complementary accession number were adapted from prior literature, where sequences were referenced but lacked an accession number. [file Table_1.docx]

Supplementary Material

**Supplementary Table S1:** Summary of odorant binding proteins (OBPs) used in the creation of the phylogenetic trees (Figure 2). Sequences without a complementary accession number were adapted from prior literature, where sequences were referenced but lacked an accession number.

| **OBP Name** | **Species** | **Accession number** |
| --- | --- | --- |
| AmelOBP1 | *Apis melifera* | AF393494 |
| AmelOBP2 | *Apis melifera* | AF393493 |
| AmelOBP3_I1 | *Apis melifera* | DQ435324 |
| AmelOBP3_I2 | *Apis melifera* | ABD92639.1 |
| AmelOBP4 | *Apis melifera* | AF393495 |
| AmelOBP5 | *Apis melifera* | AF393497 |
| AmelOBP6 | *Apis melifera* | AF393496 |
| AmelOBP7 | *Apis melifera* | DQ435325 |
| AmelOBP8_I1 | *Apis melifera* | AF339140 |
| AmelOBP8_I2 | *Apis melifera* | AF339140_1 |
| AmelOBP8_2 | *Apis melifera* | AF339140 |
| AmelOBP9 | *Apis melifera* | DQ435326 |
| AmelOBP10 | *Apis melifera* | DQ435327 |
| AmelOBP11 | *Apis melifera* | DQ435328 |
| AmelOBP12_1 | *Apis melifera* | DQ435329 |
| AmelOBP12_2 | *Apis melifera* | ABD92644.1 |
| AmelOBP13 | *Apis melifera* | DQ435330 |
| AmelOBP14 | *Apis melifera* | DQ435331 |
| AmelOBP15 | *Apis melifera* | DQ435332 |
| AmelOBP16 | *Apis melifera* | DQ435333 |
| AmelOBP17 | *Apis melifera* | DQ435334 |
| AmelOBP18 | *Apis melifera* | NP_001035317 |
| AmelOBP19 | *Apis melifera* | DQ435336 |
| AmelOBP20 | *Apis melifera* | DQ435337 |
| AmelOBP21 | *Apis melifera* | DQ435338 |
| BterOBP1JOI | *Bombus terrestris* | XP_003393962 |
| BterOBP2 | *Bombus terrestris* | XP_003397921.1 |
| BterOBP3 | *Bombus terrestris* | XP_003397912.1 |
| BterOBP4 | *Bombus terrestris* | XP_003397906.1 |
| BterOBP5 | *Bombus terrestris* | XP_003397877.1 |
| BterOBP6JOI | *Bombus terrestris* | XP_003397894.1 |
| BterOBP7 | *Bombus terrestris* | N/A |
| BterOBP8 | *Bombus terrestris* | XP_003397865.1 |
| BterOBP9 | *Bombus terrestris* | XP_003397885.1 |
| BterOBP10 | *Bombus terrestris* | XP_003401479.1 |
| BterOBP11 | *Bombus terrestris* | XP_003401480.1 |
| BterOBP12 | *Bombus terrestris* | XP_003402792.1 |
| BterOBP13 | *Bombus terrestris* | XP_003401135.1 |
| BterOBP14 | *Bombus terrestris* | XP_003398556.1 |
| BterOBP15 | *Bombus terrestris* | XP_003398555.1 |
| BterOBP16 | *Bombus terrestris* | XP_003399482.1 |
| HoblOBP1 | *Holotrichia oblita* | ACX32050.2 |
| HoblOBP2 | *Holotrichia oblita* | ACX32049.2 |
| HoblOBP3 | *Holotrichia oblita* | ADX96030.1 |
| HoblOBP4 | *Holotrichia oblita* | ADX96031.1 |
| HoblOBP5 | *Holotrichia oblita* | AZK90209.1 |
| HoblOBP6 | *Holotrichia oblita* | AZK90210.1 |
| HoblOBP7 | *Holotrichia oblita* | AZK90211.1 |
| HoblOBP8 | *Holotrichia oblita* | AZK90212.1 |
| HoblOBP9 | *Holotrichia oblita* | AZK90213.1 |
| HoblOBP10 | *Holotrichia oblita* | AZK90214.1 |
| HoblOBP11 | *Holotrichia oblita* | AZK90215.1 |
| HoblOBP12 | *Holotrichia oblita* | AZK90216.1 |
| HoblOBP13 | *Holotrichia oblita* | AZK90217.1 |
| HoblOBP14 | *Holotrichia oblita* | AZK90218.1 |
| HoblOBP15 | *Holotrichia oblita* | AZK90219.1 |
| HoblOBP16 | *Holotrichia oblita* | AZK90220.1 |
| HoblOBP17 | *Holotrichia oblita* | AZL87159.1 |
| HoblOBP18 | *Holotrichia oblita* | AZL87160.1 |
| HoblOBP19 | *Holotrichia oblita* | AZL87161.1 |
| HoblOBP20 | *Holotrichia oblita* | AZL87162.1 |
| HoblOBP21 | *Holotrichia oblita* | AZL87163.1 |
| HoblOBP22 | *Holotrichia oblita* | AZL87164.1 |
| HoblOBP23 | *Holotrichia oblita* | AZL87165.1 |
| HoblOBP24 | *Holotrichia oblita* | AZL87166.1 |
| HoblOBP25 | *Holotrichia oblita* | AZL87167.1 |
| HoblOBP26 | *Holotrichia oblita* | AZL87168.1 |
| HoblOBP27 | *Holotrichia oblita* | AZL87169.1 |
| HoblOBP28 | *Holotrichia oblita* | AZL87170.1 |
| HparOBP1 | *Holotrichia parallela* | GQ395804 |
| HparOBP2 | *Holotrichia parallela* | JF422903 |
| HparOBP20 | *Holotrichia parallela* | KR733566.1 |
| HparOBP49 | *Holotrichia parallela* | KR733548.1 |
| NvitOBP1 | *Nasonia vitripennis* | HE578186 |
| NvitOBP2 | *Nasonia vitripennis* | HE578187 |
| NvitOBP3 | *Nasonia vitripennis* | HE578188 |
| NvitOBP4 | *Nasonia vitripennis* | HE578189 |
| NvitOBP5 | *Nasonia vitripennis* | HE578190 |
| NvitOBP6 | *Nasonia vitripennis* | HE578191 |
| NvitOBP7 | *Nasonia vitripennis* | HE578192 |
| NvitOBP8 | *Nasonia vitripennis* | HE578193 |
| NvitOBP9 | *Nasonia vitripennis* | HE578194 |
| NvitOBP10 | *Nasonia vitripennis* | HE578195 |
| NvitOBP11 | *Nasonia vitripennis* | HE578196 |
| NvitOBP12 | *Nasonia vitripennis* | HE578197 |
| NvitOBP13 | *Nasonia vitripennis* | HE578198 |
| NvitOBP14 | *Nasonia vitripennis* | HE578199 |
| NvitOBP15 | *Nasonia vitripennis* | HE578200 |
| NvitOBP16 | *Nasonia vitripennis* | HE578201 |
| NvitOBP17 | *Nasonia vitripennis* | HE578202 |
| NvitOBP18 | *Nasonia vitripennis* | HE578203 |
| NvitOBP19 | *Nasonia vitripennis* | HE578204 |
| NvitOBP20 | *Nasonia vitripennis* | HE578205 |
| NvitOBP21 | *Nasonia vitripennis* | HE578206 |
| NvitOBP22 | *Nasonia vitripennis* | HE578207 |
| NvitOBP23 | *Nasonia vitripennis* | HE578208 |
| NvitOBP24 | *Nasonia vitripennis* | HE578209 |
| NvitOBP25 | *Nasonia vitripennis* | HE578210 |
| NvitOBP26 | *Nasonia vitripennis* | HE578211 |
| NvitOBP27 | *Nasonia vitripennis* | HE578212 |
| NvitOBP28 | *Nasonia vitripennis* | HE578213 |
| NvitOBP29 | *Nasonia vitripennis* | HE578214 |
| NvitOBP30 | *Nasonia vitripennis* | HE578215 |
| NvitOBP31 | *Nasonia vitripennis* | HE578216 |
| NvitOBP32 | *Nasonia vitripennis* | HE578217 |
| NvitOBP33 | *Nasonia vitripennis* | HE578218 |
| NvitOBP34 | *Nasonia vitripennis* | HE578219 |
| NvitOBP35 | *Nasonia vitripennis* | HE578220 |
| NvitOBP36 | *Nasonia vitripennis* | HE578221 |
| NvitOBP37 | *Nasonia vitripennis* | HE578222 |
| NvitOBP38 | *Nasonia vitripennis* | HE578223 |
| NvitOBP39 | *Nasonia vitripennis* | HE578224 |
| NvitOBP40 | *Nasonia vitripennis* | HE578225 |
| NvitOBP41 | *Nasonia vitripennis* | HE578226 |
| NvitOBP42 | *Nasonia vitripennis* | HE578227 |
| NvitOBP43 | *Nasonia vitripennis* | HE578228 |
| NvitOBP44 | *Nasonia vitripennis* | HE578229 |
| NvitOBP45 | *Nasonia vitripennis* | HE578230 |
| NvitOBP46 | *Nasonia vitripennis* | HE578231 |
| NvitOBP47 | *Nasonia vitripennis* | HE578232 |
| NvitOBP48 | *Nasonia vitripennis* | HE578233 |
| NvitOBP49 | *Nasonia vitripennis* | HE578234 |
| NvitOBP50 | *Nasonia vitripennis* | HE578235 |
| NvitOBP51 | *Nasonia vitripennis* | HE578236 |
| NvitOBP52 | *Nasonia vitripennis* | HE578237 |
| NvitOBP53 | *Nasonia vitripennis* | HE578238 |
| NvitOBP54 | *Nasonia vitripennis* | HE578239 |
| NvitOBP55 | *Nasonia vitripennis* | HE578240 |
| NvitOBP56 | *Nasonia vitripennis* | HE578241 |
| NvitOBP57 | *Nasonia vitripennis* | HE578242 |
| NvitOBP58 | *Nasonia vitripennis* | HE578243 |
| NvitOBP59 | *Nasonia vitripennis* | HE578244 |
| NvitOBP60 | *Nasonia vitripennis* | HE578245 |
| NvitOBP61 | *Nasonia vitripennis* | HE578246 |
| NvitOBP62 | *Nasonia vitripennis* | HE578247 |
| NvitOBP63 | *Nasonia vitripennis* | HE578248 |
| NvitOBP64 | *Nasonia vitripennis* | HE578249 |
| NvitOBP65 | *Nasonia vitripennis* | HE578250 |
| NvitOBP66 | *Nasonia vitripennis* | HE578251 |
| NvitOBP67 | *Nasonia vitripennis* | HE578252 |
| NvitOBP68 | *Nasonia vitripennis* | HE578253 |
| NvitOBP69 | *Nasonia vitripennis* | HE578254 |
| NvitOBP70 | *Nasonia vitripennis* | HE578255 |
| NvitOBP71 | *Nasonia vitripennis* | HE578256 |
| NvitOBP72 | *Nasonia vitripennis* | HE578257 |
| NvitOBP73 | *Nasonia vitripennis* | HE578258 |
| NvitOBP74 | *Nasonia vitripennis* | HE578259 |
| NvitOBP75 | *Nasonia vitripennis* | HE578260 |
| NvitOBP76 | *Nasonia vitripennis* | HE578261 |
| NvitOBP77 | *Nasonia vitripennis* | HE578262 |
| NvitOBP78 | *Nasonia vitripennis* | HE578263 |
| NvitOBP79 | *Nasonia vitripennis* | HE578264 |
| NvitOBP80 | *Nasonia vitripennis* | HE578265 |
| NvitOBP81 | *Nasonia vitripennis* | HE578266 |
| NvitOBP82 | *Nasonia vitripennis* | HE578267 |
| NvitOBP83 | *Nasonia vitripennis* | HE578268 |
| NvitOBP84 | *Nasonia vitripennis* | HE578269 |
| NvitOBP85 | *Nasonia vitripennis* | HE578270 |
| NvitOBP86 | *Nasonia vitripennis* | HE578271 |
| NvitOBP87 | *Nasonia vitripennis* | HE578272 |
| NvitOBP88 | *Nasonia vitripennis* | HE578273 |
| NvitOBP89 | *Nasonia vitripennis* | HE578274 |
| NvitOBP90 | *Nasonia vitripennis* | HE578275 |
| NvitOBP91 | *Nasonia vitripennis* | HE578276 |
| NvitOBP92 | *Nasonia vitripennis* | HE578277 |
| NvitOBP93 | *Nasonia vitripennis* | HE578278 |

**Apis florea* (AfloOBP) sequences adapted from: Mam B, Karpe SD, Sowdhamini R. Minus-C subfamily has diverged from Classic odorant-binding proteins in honeybees. Apidologie. 2023;54(1):16.

**Lepidopteran sequences adapted from: Vogt RG, Große-Wilde E, Zhou JJ. The Lepidoptera Odorant Binding Protein gene family: Gene gain and loss within the GOBP/PBP complex of moths and butterflies. Insect Biochem Mol Biol. 2015;62:142-53.

****Leptinotarsa decemlineata* (LdecOBP) adapted from: Schoville SD, Chen YH, Andersson MN, Benoit JB, Bhandari A, Bowsher JH, et al. A model species for agricultural pest genomics: the genome of the Colorado potato beetle, *Leptinotarsa decemlineata* (Coleoptera: Chrysomelidae). Sci Rep. 2018;8:18.

*****Tribolium castaneum* (TcasOBP) adapted from: Dippel S, Oberhofer G, Kahnt J, Gerischer L, Opitz L, Schachtner J, et al. Tissue-specific transcriptomics, chromosomal localization, and phylogeny of chemosensory and odorant binding proteins from the red flour beetle *Tribolium castaneum* reveal subgroup specificities for olfaction or more general functions. BMC Genomics. 2014;15:14.

**Supplementary references for Tables 1 and 2:**

1. Robertson HM, Baits RL, Walden KKO, Wada-Katsumata A, Schal C. Enormous expansion of the chemosensory gene repertoire in the omnivorous German cockroach *Blattella germanica*. Journal of Experimental Zoology Part B, Molecular and Developmental Evolution. 2018;330(5):265-78.

2. He P, Li ZQ, Zhang YF, Chen L, Wang J, Xu L, et al. Identification of odorant-binding and chemosensory protein genes and the ligand affinity of two of the encoded proteins suggest a complex olfactory perception system in *Periplaneta americana*. Insect Molecular Biology. 2017;26(6):687-701.

3. McKenna DD, Scully ED, Pauchet Y, Hoover K, Kirsch R, Geib SM, et al. Genome of the Asian longhorned beetle (*Anoplophora glabripennis*), a globally significant invasive species, reveals key functional and evolutionary innovations at the beetle–plant interface. Genome Biol. 2016;17(1):227.

4. Andersson MN, Grosse-Wilde E, Keeling CI, Bengtsson JM, Yuen MMS, Li M, et al. Antennal transcriptome analysis of the chemosensory gene families in the tree killing bark beetles, *Ips typographus* and *Dendroctonus ponderosae* (Coleoptera: Curculionidae: Scolytinae). BMC Genomics. 2013;14:16.

5. Yin J, Wang CQ, Fang CQ, Zhang S, Cao YZ, Li KB, et al. Functional characterization of odorant-binding proteins from the scarab beetle *Holotrichia oblita* based on semiochemical-induced expression alteration and gene silencing. Insect Biochem Mol Biol. 2019;104:11-9.

6. Ju Q, Li X, Jiang XJ, Qu MJ, Guo XQ, Han ZJ, et al. Transcriptome and tissue-specific expression analysis of OBP and CSP genes in the dark balck charfer. Arch Insect Biochem Physiol. 2014;87(4):177-200.

7. Schoville SD, Chen YH, Andersson MN, Benoit JB, Bhandari A, Bowsher JH, et al. A model species for agricultural pest genomics: the genome of the Colorado potato beetle, *Leptinotarsa decemlineata* (Coleoptera: Chrysomelidae). Scientific reports. 2018;8:18.

8. Liu S, Rao XJ, Li MY, Feng MF, He MZ, Li SG. Identification of candidate chemosensory genes in the antennal transcriptome of *Tenebrio molitor* (Coleoptera: Tenebrionidae). Comp Biochem Physiol D-Genomics Proteomics. 2015;13:44-51.

9. Dippel S, Oberhofer G, Kahnt J, Gerischer L, Opitz L, Schachtner J, et al. Tissue-specific transcriptomics, chromosomal localization, and phylogeny of chemosensory and odorant binding proteins from the red flour beetle *Tribolium castaneum* reveal subgroup specificities for olfaction or more general functions. BMC Genomics. 2014;15:14.

10. Richards S, Gibbs RA, Weinstock GM, Brown SJ, Denell R, Beeman RW, et al. The genome of the model beetle and pest *Tribolium castaneum*. Nature. 2008;452(7190):949-55.

11. Manoharan M, Ng Fuk Chong M, Vaïtinadapoulé A, Frumence E, Sowdhamini R, Offmann B. Comparative genomics of odorant binding proteins in *Anopheles gambiae*, *Aedes aegypti*, and *Culex quinquefasciatus*. Genome Biology and Evolution. 2013;5(1):163-80.

12. Zafar Z, Fatima S, Bhatti MF, Shah FA, Saud Z, Butt TM. Odorant Binding Proteins (OBPs) and Odorant Receptors (ORs) of *Anopheles stephensi*: Identification and comparative insights. PloS One. 2022;17(3):e0265896.

13. Hekmat-Scafe DS, Scafe CR, McKinney AJ, Tanouye MA. Genome-wide analysis of the odorant-binding protein gene family in *Drosophila melanogaster*. Genome research. 2002;12(9):1357-69.

14. Graham LA, Davies PL. The odorant-binding proteins of *Drosophila melanogaster*: annotation and characterization of a divergent gene family. Gene. 2002;292(1-2):43-55.

15. Rondón JJ, Moreyra NN, Pisarenco VA, Rozas J, Hurtado J, Hasson E. Evolution of the odorant-binding protein gene family in *Drosophila*. Frontiers in Ecology and Evolution. 2022;10.

16. Zhou JJ, Vieira FG, He XL, Smadja C, Liu R, Rozas J, et al. Genome annotation and comparative analyses of the odorant-binding proteins and chemosensory proteins in the pea aphid *Acyrthosiphon pisum*. Insect Mol Biol. 2010;19:113-22.

17. Gu SH, Wang SP, Zhang XY, Wu KM, Guo YY, Zhou JJ, et al. Identification and tissue distribution of odorant binding protein genes in the lucerne plant bug *Adelphocoris lineolatus* (Goeze). Insect Biochem Mol Biol. 2011;41(4):254-63.

18. Zeng Y, Yang YT, Wu QJ, Wang SL, Xie W, Zhang YJ. Genome-wide analysis of odorant-binding proteins and chemosensory proteins in the sweet potato whitefly, Bemisia tabaci. Insect science. 2019;26(4):620-34.

19. Li JB, Yin MZ, Yao WC, Ma S, Dewer Y, Liu XZ, et al. Genome-wide analysis of odorant-binding proteins and chemosensory proteins in the bean bug *Riptortus pedestris*. Frontiers in physiology. 2022;13:949607.

20. Song YQ, Sun HZ, Du J. Identification and tissue distribution of chemosensory protein and odorant binding protein genes in *Tropidothorax elegans* Distant (Hemiptera: Lygaeidae). Scientific reports. 2018;8(1):7803.

21. Jiang X, Qin Y, Jiang J, Xu Y, Francis F, Fan J, et al. Spatial expression analysis of odorant binding proteins in both sexes of the aphid Parasitoid *Aphidius gifuensis* and their ligand binding properties. Frontiers in Physiology. 2022;13:877133.

22. Mam B, Karpe SD, Sowdhamini R. Minus-C subfamily has diverged from Classic odorant-binding proteins in honeybees. Apidologie. 2023;54(1):16.

23. Foret S, Maleszka R. Function and evolution of a gene family encoding odorant binding-like proteins in a social insect, the honey bee (*Apis mellifera*). Genome Research. 2006;16(11):1404-13.

24. Sadd BM, Barribeau SM, Bloch G, de Graaf DC, Dearden P, Elsik CG, et al. The genomes of two key bumblebee species with primitive eusocial organization. Genome Biology. 2015;16(1):76.

25. Xiao JH, Yue Z, Jia LY, Yang XH, Niu LH, Wang Z, et al. Obligate mutualism within a host drives the extreme specialization of a fig wasp genome. Genome Biology. 2013;14(12):R141.

26. Wang N, Wang NX, Niu LM, Bian SN, Xiao JH, Huang DW. Odorant-binding protein (OBP) genes affect host specificity in a fig-pollinator mutualistic system. Insect Molecular Biology. 2014;23(5):621-31.

27. Shi M, Wang Z, Ye X, Xie H, Li F, Hu X, et al. The genomes of two parasitic wasps that parasitize the diamondback moth. BMC Genomics. 2019;20(1):893.

28. Zhou YN, Xie S, Chen JN, Wang ZH, Yang P, Zhou SC, et al. Expression and functional characterization of odorant-binding protein genes in the endoparasitic wasp *Cotesia vestalis*. Insect Science. 2021;28(5):1354-68.

29. Vieira FG, Foret S, He XL, Rozas J, Field LM, Zhou JJ. Unique features of odorant-binding proteins of the parasitoid wasp *Nasonia vitripennis* revealed by genome annotation and comparative analyses. PloS One. 2012;7(8):11.

30. Gong DP, Zhang HJ, Zhao P, Xia QY, Xiang ZH. The odorant binding protein gene family from the genome of silkworm, *Bombyx mori*. BMC Genomics. 2009;10:332.

31. Vogt RG, Große-Wilde E, Zhou JJ. The Lepidoptera Odorant Binding Protein gene family: Gene gain and loss within the GOBP/PBP complex of moths and butterflies. Insect Biochem Mol Biol. 2015;62:142-53.

32. Cai LJ, Zheng LS, Huang YP, Xu W, You MS. Identification and characterization of odorant binding proteins in the diamondback moth, *Plutella xylostella*. Insect Science. 2021;28(4):987-1004.

33. Jia C, Mohamed A, Cattaneo AM, Huang X, Keyhani NO, Gu M, et al. Odorant-binding proteins and chemosensory proteins in *Spodoptera frugiperda*: from genome-wide identification and developmental stage-related expression analysis to the perception of host plant odors, sex pheromones, and insecticides. International Journal of Molecular Sciences. 2023;24(6).

34. Guo W, Ren D, Zhao L, Jiang F, Song J, Wang X, et al. Identification of Odorant-binding proteins (OBPs) and functional analysis of phase-related OBPs in the migratory locust. Frontiers in physiology. 2018;9:984.

35. Jiang XC, Krieger J, Breer H, Pregitzer P. Distinct subfamilies of odorant binding proteins in locust (Orthoptera, Acrididae): molecular evolution, structural variation, and sensilla-specific expression. Front Physiol. 2017;8:15.

36. Liu Y, Luo Y, Du L, Ban L. Antennal transcriptome analysis of olfactory genes and characterization of odorant binding proteins in *Odontothrips loti* (Thysanoptera: Thripidae). International Journal of Molecular Sciences. 2023;24(6).

37. Lartigue A, Gruez A, Spinelli S, Riviere S, Brossut R, Tegoni M, et al. The crystal structure of a cockroach pheromone-binding protein suggests a new ligand binding and release mechanism. J Biol Chem. 2003;278(32):30213-8.

38. Rivière S, Lartigue A, Quennedey B, Campanacci V, Farine JP, Tegoni M, et al. A pheromone-binding protein from the cockroach *Leucophaea maderae*: cloning, expression and pheromone binding. The Biochemical journal. 2003;371(Pt 2):573-9.

39. Rothemund S, Liou YC, Davies PL, Krause E, Sonnichsen FD. A new class of hexahelical insect proteins revealed as putative carriers of small hydrophobic ligands. Struct Fold Des. 1999;7(11):1325-32.

40. Leite NR, Krogh R, Xu W, Ishida Y, Iulek J, Leal WS, et al. Structure of an odorant-binding protein from the mosquito *Aedes aegypti* suggests a binding pocket covered by a pH-sensitive "Lid". PloS One. 2009;4(11):7.

41. Wang J, Murphy EJ, Nix JC, Jones DNM. Aedes aegypti Odorant Binding Protein 22 selectively binds fatty acids through a conformational change in its C-terminal tail. Sci Rep. 2020;10(1):15.

42. Wogulis M, Morgan T, Ishida Y, Leal WS, Wilson DK. The crystal structure of an odorant binding protein from *Anopheles gambiae*: Evidence for a common ligand release mechanism. Biochem Biophys Res Commun. 2006;339(1):157-64.

43. Biessmann H, Andronopoulou E, Biessmann MR, Douris V, Dimitratos SD, Eliopoulos E, et al. The *Anopheles gambiae* odorant binding protein 1 (AgamOBP1) mediates indole recognition in the antennae of female mosquitoes. PLoS One. 2010;5(3):8.

44. Tsitsanou KE, Thireou T, Drakou CE, Koussis K, Keramioti MV, Leonidas DD, et al. *Anopheles gambiae* odorant binding protein crystal complex with the synthetic repellent DEET: implications for structure-based design of novel mosquito repellents. Cell Mol Life Sci. 2012;69(2):283-97.

45. Murphy EJ, Booth JC, Davrazou F, Port AM, Jones DNM. Interactions of *Anopheles gambiae* Odorant-binding proteins with a human-derived repellent implications for the mode of action of N,N-diethyl-3-methylbenzamide (DEET). J Biol Chem. 2013;288(6):4475-85.

46. Lagarde A, Spinelli S, Tegoni M, He XL, Field L, Zhou JJ, et al. The crystal structure of odorant binding protein 7 from *Anopheles gambiae* exhibits an outstanding adaptability of its binding site. Journal of Molecular Biology. 2011;414(3):401-12.

47. Ziemba BP, Murphy EJ, Edlin HT, Jones DNM. A novel mechanism of ligand binding and release in the odorant binding protein 20 from the malaria mosquito *Anopheles gambiae*. Protein Sci. 2013;22(1):11-21.

48. Lagarde A, Spinelli S, Qiao HL, Tegoni M, Pelosi P, Cambillau C. Crystal structure of a novel type of odorant-binding protein from *Anopheles gambiae*, belonging to the C-plus class. Biochem J. 2011; 437:423-30.

49. Tsitsanou KE, Drakou CE, Thireou T, Gruber AV, Kythreoti G, Azem A, et al. Crystal and solution studies of the "Plus-C" Odorant-binding protein 48 from *Anopheles gambiae* control of binding specificity through three-dimentional domain swapping. Journal of Biological Chemistry. 2013; 288(46):33427-38.

50. Mao Y, Xu XZ, Xu W, Ishida Y, Leal WS, Ames JB, et al. Crystal and solution structures of an odorant-binding protein from the southern house mosquito complexed with an oviposition pheromone. Proc Natl Acad Sci U S A. 2010;107(44):19102-7.

51. Pelletier J, Guidolin A, Syed Z, Cornel AJ, Leal WS. Knockdown of a mosquito odorant-binding protein involved in the sensitive detection of oviposition attractants. J Chem Ecol. 2010;36(3):245-8.

52. Gonzalez D, Rihani K, Neiers F, Poirier N, Fraichard S, Gotthard G, et al. The *Drosophila* odorant-binding protein 28a is involved in the detection of the floral odour beta-ionone. Cellular and Molecular Life Sciences. 2020;77(13):2565-77.

53. Kruse SW, Zhao R, Smith DP, Jones DNM. Structure of a specific alcohol-binding site defined by the odorant binding protein LUSH from *Drosophila melanogaster*. Nat Struct Biol. 2003;10(9):694-700.

54. Xu PX, Atkinson R, Jones DNM, Smith DP. *Drosophila* OBP LUSH is required for activity of pheromone-sensitive neurons. Neuron. 2005;45(2):193-200.

55. Thode AB, Kruse SW, Nix JC, Jones DNM. The role of multiple hydrogen-bonding groups in specific alcohol binding sites in proteins: Insights from structural studies of LUSH. J Mol Biol. 2008;376(5):1360-76.

56. Northey T, Venthur H, De Biasio F, Chauviac FX, Cole A, Ribeiro K, et al. Crystal Structures and Binding Dynamics of Odorant-Binding Protein 3 from two aphid species *Megoura viciae* and *Nasonovia ribisnigri*. Sci Rep. 2016;6:13.

57. Danty E, Briand L, Michard-Vanhee C, Perez V, Arnold G, Gaudemer O, et al. Cloning and expression of a queen pheromone-binding protein in the honeybee: an olfactory-specific, developmentally regulated protein. J Neurosci. 1999;19(17):7468-75.

58. Lartigue A, Gruez A, Briand L, Blon F, Bezirard V, Walsh M, et al. Sulfur single-wavelength anomalous diffraction crystal structure of a pheromone-binding protein from the honeybee *Apis mellifera* L. J Biol Chem. 2004;279(6):4459-64.

59. Pesenti ME, Spinelli S, Bezirard V, Briand L, Pernollet JC, Tegoni M, et al. Structural basis of the honey bee PBP pheromone and pH-induced conformational change. Journal of molecular biology. 2008;380(1):158-69.

60. Pesenti ME, Spinelli S, Bezirard V, Briand L, Pernollet JC, Campanacci V, et al. Queen bee pheromone binding protein pH-induced domain swapping favors pheromone release. J Mol Biol. 2009;390(5):981-90.

61. Danty E, MichardVanhee C, Huet JC, Genecque E, Pernollet JC, Masson C. Biochemical characterization, molecular cloning and localization of a putative odorant-binding protein in the honey bee *Apis mellifera* L. (Hymenoptera: Apidea). FEBS Lett. 1997;414(3):595-8.

62. Briand L, Nespoulous C, Huet JC, Takahashi M, Pernollet JC. Ligand binding and physico-chemical properties of ASP2, a recombinant odorant-binding protein from honeybee (*Apis mellifera* L.). European journal of biochemistry. 2001;268(3):752-60.

63. Spinelli S, Lagarde A, Iovinella I, Legrand P, Tegoni M, Pelosi P, et al. Crystal structure of *Apis mellifera* OBP14, a C-minus odorant-binding protein, and its complexes with odorant molecules. Insect Biochem Mol Biol. 2012;42(1):41-50.

64. Xu X, Xu W, Rayo J, Ishida Y, Leal WS, Ames JB. NMR structure of navel orangeworm moth pheromone-binding protein (AtraPBP1): implications for pH-sensitive pheromone detection. Biochemistry. 2010;49(7):1469-76.

65. Xu W, Xu X, Leal WS, Ames JB. Extrusion of the C-terminal helix in navel orangeworm moth pheromone-binding protein (AtraPBP1) controls pheromone binding. Biochemical and Biophysical Research Communications. 2011;404(1):335-8.

66. Mohanty S, Zubkov S, Gronenborn AM. The solution NMR structure of *Antheraea polyphemus* PBP provides new insight into pheromone recognition by pheromone-binding proteins. J Mol Biol. 2004;337(2):443-51.

67. Zubkov S, Gronenborn AM, Byeon IJ, Mohanty S. Structural consequences of the pH-induced conformational switch in *A. polyphemus* pheromone-binding protein: mechanisms of ligand release. J Mol Biol. 2005;354(5):1081-90.

68. Damberger FF, Ishida Y, Leal WS, Wüthrich K. Structural basis of ligand binding and release in insect pheromone-binding proteins: NMR structure of *Antheraea polyphemus* PBP1 at pH 4.5. Journal of molecular biology. 2007;373(4):811-9.

69. Forstner M, Breer H, Krieger J. A receptor and binding protein interplay in the detection of a distinct pheromone component in the silkmoth *Antheraea polyphemus*. Int J Biol Sci. 2009;5(7):745-57.

70. Sandler BH, Nikonova L, Leal WS, Clardy J. Sexual attraction in the silkworm moth: structure of the pheromone-binding-protein-bombykol complex. Chem Biol. 2000;7(2):143-51.

71. Lautenschlager C, Leal WS, Clardy J. *Bombyx mori* pheromone-binding protein binding nonpheromone ligands: implications for pheromone recognition. Structure (London, England : 1993). 2007;15(9):1148-54.

72. Shiota Y, Sakurai T, Daimon T, Mitsuno H, Fujii T, Matsuyama S, et al. *In vivo* functional characterisation of pheromone binding protein-1 in the silkmoth, *Bombyx mori*. Sci Rep. 2018;8:8.

73. Zhou JJ, Robertson G, He XL, Dufour S, Hooper AM, Pickett JA, et al. Characterisation of *Bombyx mori* odorant-binding proteins reveals that a general odorant-binding protein discriminates between sex pheromone components. J Mol Biol. 2009;389(3):529-45.

74. He XL, Tzotzos G, Woodcock C, Pickett JA, Hooper T, Field LM, et al. Binding of the general odorant binding protein of *Bombyx mori* BmorGOBP2 to the moth sex pheromone components. J Chem Ecol. 2010;36(12):1293-305.

75. Hamiaux C, Carraher C, Lofstedt C, Corcoran JA. Crystal structure of *Epiphyas postvittana* pheromone binding protein 3. Sci Rep. 2020;10(1):11.

76. Dong K, Duan HX, Liu JT, Sun L, Gu SH, Yang RN, et al. Key site residues of pheromone-binding protein 1 involved in interacting with sex pheromone components of *Helicoverpa armigera*. Sci Rep. 2017;7:9.

77. Zheng JG, Yang MT, Dong K, Zhang JB, Wang HL, Xie MJ, et al. Structural insights into the ligand-binding and -releasing mechanism of *Helicoverpa armigera* pheromone-binding protein PBP1. Int J Mol Sci. 2022;23(3):11.

78. Terrado M, Okon M, McIntosh LP, Plettner E. Ligand- and pH-induced structural transition of Gypsy moth *Lymantria dispar* pheromone-binding protein 1 (LdisPBP1). Biochemistry. 2020;59(37):3411-26.

79. Li ZQ, Zhang S, Cai XM, Luo JY, Dong SL, Cui JJ, et al. Three odorant binding proteins may regulate the behavioural response of *Chrysopa pallens* to plant volatiles and the aphid alarm pheromone (E)-β-farnesene. Insect Molecular Biology. 2017;26(3):255-65.

80. Li TT, Liu WC, Zhu J, Yang YH, Ma C, Lu C, et al. Crystal structure and ligand identification of odorant binding protein 4 in the natural predator *Chrysopa pallens*. Int J Biol Macromol. 2019;141:1004-12.

81. Zheng JG, Li JR, Han L, Wang Y, Wu W, Qi XX, et al. Crystal structure of the *Locusta migratoria* odorant binding protein. Biochem Biophys Res Commun. 2015;456(3):737-42.
